# AppReminders – a pilot feasibility randomized controlled trial of a memory aid app for people with acquired brain injury

**DOI:** 10.1080/09602011.2023.2220969

**Published:** 2023-06-13

**Authors:** Matthew Jamieson, Heather McClelland, Nicola Goudie, Jean McFarlane, Breda Cullen, Marilyn Lennon, Stephen Brewster, Bethany Stanley, Alex McConnachie, Jonathan Evans

**Affiliations:** a School of Health and Wellbeing, University of Glasgow, Glasgow, UK; b Human Computer Interaction, Department of Computing Science, University of Glasgow, Glasgow, UK; c Community Treatment Centre for Brain Injury, NHS Greater Glasgow and Clyde, Glasgow, UK; d Acquired Brain Injury (ABI) Service, West Dunbartonshire HSPC, Dumbarton, UK; e Computer and Information Science, University of Strathclyde, Glasgow, UK; f Robertson Centre for Biostatistics, University of Glasgow, Glasgow, UK

**Keywords:** Assistive technology, Acquired brain injury, Memory rehabilitation, Feasibility trial

## Abstract

Mobile phone reminding apps can be used by people with acquired brain injury (ABI) to compensate for memory impairments. This pilot feasibility trial aimed to establish the feasibility of a randomized controlled trial comparing reminder apps in an ABI community treatment setting. Adults with ABI and memory difficulty who completed the three-week baseline were randomized (*n* = 29) and allocated to Google Calendar or ApplTree app. Those who attended an intervention session (*n* = 21) watched a 30-minute video tutorial of the app then completed reminder setting assignments to ensure they could use the app. Guidance was given if needed from a clinician or researcher. Those who passed the app assignments (*n* = 19) completed a three-week follow up. Recruitment was lower than target (*n* = 50), retention rate was 65.5%, adherence rate was 73.7%. Qualitative feedback highlighted issues that may impact usability of reminding apps introduced within community brain injury rehabilitation. Feasibility results indicate a full trial would require 72 participants to demonstrate the minimally clinically important efficacy difference between apps, should a difference exist. Most participants (19 of 21) given an app could learn to use it with the short tutorial. Design features implemented in ApplTree have potential to improve the uptake and utility of reminding apps.

## Introduction

Neurological impairment and acquired brain injury is a leading cause of disability. It is estimated that 387 million people (4.9% of the worldwide population) will have a neurological impairment due to an acquired brain injury (ABI) (ABI includes stroke) or a degenerative disease by 2030 (WHO, 2020). There were 480,652 hospital admissions for brain injury and stroke in the UK in 2016/17 (Headway, 2020). The prevalence of survivors of brain injury with a disability in the UK is thought to be 100–150 per 100,000 (King & Dean, 2009). People with brain injury commonly experience difficulties with memory, concentration, attention and judgement, meaning that important everyday actions and tasks are not carried out or not completed, limiting the ability to live independently.

Technologies that send timely prompts to people about everyday activities are an effective, low-cost solution to support people with cognitive impairments after brain injury. A systematic review and meta-analysis (Jamieson et al., 2014) found that prompting technology that prompts people to carry out intended tasks improves memory performance for people with memory difficulties vs. practice as usual or a paper diary/calendar (d = 1.27, *n* = 147). Reminding technology can also reduce the burden on caregivers (Teasdale et al., 2009). The INCOG 2.0 guidelines (Bayley et al., 2023) state that electronic reminder systems like smartphone reminders are preferrable to non-technological external memory aids as a primary strategy for compensating for severe memory impairment. A recent systematic review (Ownsworth et al., 2023) systematically reviewed the literature evaluating efficacy of electronic assistive technology to support memory for people with traumatic brain injury. They found the most support for technologies that supported retrieval of information from memory and execution of everyday tasks that needed to be remembered.

This potential positive impact will only be seen in practice if people have access to this technology (e.g., as part of their clinical rehabilitation). The positive impact of reminding technology use will be greater if people are able to use the technology independently when it is provided, and if the technology meets the individual needs of the users. Indeed, the systematic review of by Ownsworth et al. (2023) called for further research into how factors such as differences between users needs, the extent to which they use the technology independently, and different app features, influence uptake and effectiveness of these technologies.

Although technological memory aids can play an important role in brain injury rehabilitation, uptake of reminding technology is currently low. People with ABI use these aids less than the general population and less than the use of non-technological memory aids. A survey found that 79% of people with ABI (*n* = 81) used paper calendars while 38% used their mobile phone to remind them (Jamieson et al., 2017). A recent study also found more barriers to the use of assistive technology to support cognition than other types of assistive technology such as myoelectric prostheses and powered mobility devices (Widehammar et al., 2019).

Research exploring smartphone users with ABI has highlighted that memory and attention difficulties prevent people from making effective use of reminding apps (Jamieson et al., 2015; Jamieson, Cullen, et al., 2022; Wong et al., 2017). Potential barriers to uptake and use of assistive technology include difficulty initially learning to use them, forgetting or losing motivation to use them, and not being supported by caregivers (de Joode, van Boxtel, et al., 2012; Jamieson et al., 2017; Juengst et al., 2021). Training with apps can overcome the barriers to uptake and improve subsequent use (Ramirez-Hernandez et al., 2020; Svoboda et al., 2010). Recommending apps and training service users in how to use their phone or reminder app is routine practice in neuropsychological rehabilitation (Ramirez-Hernandez et al., 2020; Wong et al., 2017). However, lengthy training in inpatient or community neuropsychological rehabilitation is difficult to find time for and is expensive. Furthermore, when choosing reminding apps, clinicians turn to reminding apps available on the app store such as Google Calendar (de Joode, Proot, et al., 2012; Mcdonald et al., 2011) or the Cozi Family Organiser (Ramirez-Hernandez et al., 2022) in the absence of purpose-built technologies.

Within this rehabilitation context it has been found that apps developed for the general population can be difficult to use due to cognitive impairments common after ABI (de Joode, van Boxtel, et al., 2012; Jamieson et al., 2015). Users with ABI forget, or do not realize, they need to enter reminders (Jamieson et al., 2017). Learning to set accurate reminders may be difficult especially when the phone or app is unfamiliar to the user (Jamieson, Lennon, et al., 2022; Wong et al., 2017). A carer or member of the person’s family could set reminders for them and studies have demonstrated the efficacy of reminders set by a third-party (Evans et al., 1998; Wilson et al., 2001). However, many people with ABI would prefer to independently set reminders and learning to independently support memory is often a goal set in neuropsychological rehabilitation (Wong et al., 2017). The culmination of these barriers means that those who could benefit most from reminding technology are those for whom it is least accessible.

This paper builds on extensive previous work developing the ApplTree app – smartphone reminding software with personalizable features, specifically designed for people with brain injury to improve everyday functioning. These features include push prompts that ask “Do you need to set any reminders?” and a user interface that breaks the process of setting reminders down into small steps (Jamieson et al., 2015; Jamieson et al., 2017). While there have been several research papers that have investigated the efficacy of reminding technologies compared to practice as usual or pencil and paper alternatives (Mcdonald et al., 2011; Ramirez-Hernandez et al., 2021; Wilson et al., 2001), the literature lacks comparisons between different technologies or different apps.

The trial reported is a pilot feasibility randomized controlled trial which compared ApplTree with Google Calendar – a widely used reminding app without these features. Google Calendar has a schedule screen for viewing events and reminders. Events are set using a single screen that includes all information you may need to set a reminder. This trial comparing ApplTree and Google Calendar will underpin a large-scale Randomized Controlled Trial (RCT) to examine efficacy (see supplementary materials doc. 1 for a more detailed description of both apps). We wish to find out if such an intervention can improve the everyday memory performance of individuals with memory difficulties following ABI compared to an off-the-shelf reminding app (Google Calendar). Recruitment, participant retention and adherence data were gathered to inform a future larger scale efficacy trial of ApplTree as an intervention to support memory in people with ABI. Information about the experience of using the apps was also gathered to gain an understanding of the factors that influence the feasibility and acceptability of reminding app interventions in neuropsychological rehabilitation.

## Methods

A parallel randomized controlled feasibility trial with 1:1 allocation ratio for ApplTree (intervention) and Google Calendar (control) was undertaken. The trial was funded by a Chief Scientist Office (CSO) Translational Clinical Studies Research Committee award (TCS/18/09) and was pre-registered on ClinicalTrial.gov (NCT04551651). The trial received approval from the National Health Service (NHS) Research Ethics Committee on 25/06/19 (19/ES/0060). Recruitment took place from the beginning of July 2019 until the end of April 2021. All follow up sessions were completed by the end of August 2021. There was a 9 month break due to the Covid-19 pandemic between March 2020 and December 2020. When the study restarted, study sessions that had originally been in person were delivered remotely. The study recruitment was intended to represent one year of recruitment so that the feasibility of running this type of trial could be investigated.

### Primary objective

The primary objective was to understand the feasibility of running a randomized controlled trial investigating the impact of the ApplTree reminding app on memory performance for individuals receiving community treatment for ABI compared to an off-the-shelf alternative (Google Calendar). To understand the feasibility we were interested in recruitment (how many people will take part in the study?), retention (do those people stay in the study?) and adherence (do people taking part carry out the activities they are asked to do as part of the study?).

The following key feasibility questions were investigated.

#### Recruitment

Can 80% or above of the planned number of people be recruited into this study (randomized)?

#### Retention

Do the majority (70% or above) of participants attend the follow-up sessions until the end of the trial?

#### Adherence

Do the majority (70% or above) of participants who receive the app use it until the end of the trial? Use of the app was defined as using the app at least once in each of the three weeks of the follow-up.

The initial recruitment target of *n* = 50 was based on the estimated number of participants who would meet criteria within the study timeline. The target to recruit more than or equal to 80% of this number, retain 70% of those randomized, and for 70% of those retained to adhere to the intervention, was set based on the judgement of the clinical and research teams of what proportion of this target would illustrate feasibility for a full trial. Using these figures, the estimated number of people recruited finishing the trial and adhering to the study intervention (around 50%) matched conservative estimates of the average attrition rate that is seen in psychological interventions (Wierzbicki & Pekarik, 1993). For example, if 50 people were recruited then 35 (70% of 50) would be expected to be retained and 25 (70% of 35) would have adhered to the intervention.

### Secondary objectives

Secondary exploratory research questions were investigated using the feasibility data, neuropsychological assessment information about participants, field notes, observations and recorded feedback from participants:

1. To inform the development of a randomized controlled trial we wished to know:
  - What are the reasons for service users who meet the criteria not participating in the research, and why and when do people leave the research after they have enrolled?
  - How many people should be recruited to adequately power a future randomized controlled trial?
2. To describe and understand what influences user experiences of smartphone reminding in a brain injury community treatment setting:
  - What are people's experiences of using the memory aid apps (ApplTree and Google Calendar) provided in this study? This information was provided using a quantitative measure (the Unified Theory of Acceptance and Use of Technology (UTAUT) questionnaire) and qualitative feedback from participants.
  - Does neuropsychological profile (memory, executive function and attention ability) influence reminder app effectiveness and the need for support?

### Study population

Participants were recruited from the Community Treatment Centre for Brain Injury in Glasgow (CTCBI) (a NHS GG&C service), the West Dunbartonshire Health and Social Care Partnership Acquired Brain Injury Service (WDMCN) and a Brain Injury Rehabilitation Trust outpatient rehabilitation service in Renfrewshire (Quarriers Renfrewshire Acquired Brain Injury service – QRBIS) and Graham Anderson House (GAH). Staff from these services identified and approached eligible service users.

Inclusion criteria consisted of adults (over 18 years) with an ABI (confirmed by the service) and self or other reported memory difficulties. The services involved defined ABI as any non-progressive brain injury or damage that was acquired following impact or illness (this includes stroke). Exclusion criteria were the inability to provide informed consent for research participation, not owning a smartphone compatible with ApplTree and Google Calendar, inadequate writing or reading (English) which would impair comprehension and performance of experimental tasks and / or answering of questionnaires, the inability to verbally communicate adequately in an experimental setting, and severe physical or sensory disability which would prevent any attempt at using a typical smartphone device (e.g., paralysis of both upper limbs). The study protocol was altered after the Covid-19 pandemic to involve fully remote sessions using phone or video calls. A further exclusion criterion of not having internet access and technology at home necessary to set up video call sessions or complete the app intervention session was added for the post-Covid-19 trial restart.

#### Identification of participants and consent

The clinical teams at recruiting sites were responsible for approaching participants in the first instance. The research team were given contact details for potential participants after they had expressed interest in taking part in the study. Enrolment was completed during the first study session with a researcher and this included in-person (pre-Covid-19) or verbal over the phone (post-Covid-19) signing of the consent form which was also sent to the participant for written confirmation. The clinical team aggregated details about the age, gender, and reasons for approaching/not approaching at screening stage.

### Study design

#### Study sessions

The flowchart outlining the trial structure can be seen in Figure 2 in results section. There were 11 study sessions in total. Prior to the Covid-19 pandemic there were five face-to-face sessions and six calls. Post Covid-19 all the sessions were calls and videocalls were used where possible for the sessions that had previously been face to face. Pre-Covid the 11 study sessions, in order (with week that this session was ideally completed in) were:

1. In person (week 1): **Enrolment**, demographic questions, memory log folder given.
2. Call (week 2): **Baseline** phone call 1 to go through memory log and fill in week 2.
3. Call (week 3): **Baseline** phone call 2 to go through memory log and fill in week 3.
4. Call (week 4): **Baseline** phone call 3 phone call to go through memory log.
5. In person (weeks 1–4): **Neuropsychological tests** administered.
6. In person (week 5): **Intervention** session followed by 6 weeks of independent app use.
7. In person (week 11): **Follow-up session 1**, app experience questions, memory log folder given.
8. Call (week 12): **Follow-up** phone call 1 go through memory log and fill in week 2.
9. Call (week 13): **Follow-up** phone call 2 go through memory log and fill in week 3.
10. Call (week 14): **Follow-up** phone call 3 go through memory log.
11. In person (weeks 14–15): **Debrief**, gather data about app use and app experience questions.

While participants could complete the study in 15 weeks it often took longer due to delays between sessions. A maximum of two weeks was allowed between any of the study sessions. Participants did not need to attend the 2nd or 3rd baseline or follow-up calls for their participation in the study to continue as 1 week of baseline and follow-up data could still be used in the analysis. Once the participant had completed the baseline phase and been randomized there was no time limit to begin the intervention, although they were invited to receive the intervention as soon as randomization was completed and the intervention could be delivered by the service.

#### Randomization

Participants were randomized to ApplTree or Google Calendar after the final week of the baseline phase. Participants were randomized if they had provided at least one week of memory log and text time data during the baseline phase. Randomization was stratified by type of brain injury (traumatic brain injury (TBI) or other acquired brain injury) and recruiting service. TBI is categorized under the umbrella term ABI but includes only brain injuries that were sustained through a traumatic injury such as a fall or road traffic accident. A stratified allocation sequence was computer-generated, using the R statistical software, by the method of randomized permuted blocks of length 4. The allocation sequence (and source program, with random seed) was kept in a secure area of the Robertson Centre for Biostatistics (RCB) network, at the University of Glasgow, and was accessible only by those responsible for the development and maintenance of the randomization system. Randomization was completed by a researcher who was not blinded to intervention condition via telephone to an interactive voice response system.

### Intervention session

After randomization, the intervention session was arranged when participants received either the Google Calendar or ApplTree app. Prior to the Covid-19 pandemic, this session was in person with a clinician or rehabilitation worker from the recruiting service. Post Covid-19 this was a phone or video call session completed by either the clinician/rehabilitation worker or the researcher who was not blinded to condition.

Participants watched a tutorial video showing them how to use either Google Calendar or ApplTree on their type of phone (Android or iOS). The videos can be found on vimeo.com (https://vimeo.com/search?q = appreminders) and are also available from the corresponding author on request.

Participants were then asked to complete a reminder setting assignment where they were given assignment sheets to verify that they were able to set reminders accurately using the app. The clinician/worker or researcher running the session took a note of whether the participant had watched the video fully, any issues the person had, guidance they gave, and the scores they received for the assignments. They followed a script and checklist to ensure intervention fidelity in terms of the instructions to participants and scoring of the assignments. The assignment sheets and delivery instructions are available as supplementary materials document 2.

### Descriptive measures

Demographic information collected was age, sex, living situation, deprivation level of area they lived in (measured by the Scottish Index of Multiple Deprivation, SIMD), work or education status, length of time since injury, cause of acquired brain injury (ABI vs TBI was used in the stratification), details about the frequency of their memory aid and calendar use (see supplementary materials doc 1) and neuropsychological assessment data.

### Outcome measures

The outcome measures for the primary objectives were recruitment, retention, and adherence. Recruitment (the number of study referrals who were enrolled and randomized) and retention (follow-up study session attendance for those randomized were noted by the research team). Adherence was measured as the number of reminders the participants received from the app in each of the three weeks of the follow-up phase. This information was gathered in the final study session by either, (i) looking at the server that noted the reminders added (ApplTree participants only), (ii) asking participants to share their calendar virtually or by physically showing the app calendar screen to the researcher (Google Calendar participants only), or (iii) asking participants to describe how many reminders they had in their calendar for each day of the three follow-up weeks (Google Calendar participants only, who weren’t able or did not want to share their calendar).

The outcome measures for the secondary objectives included asking participants to keep a daily (non-electronic) memory log and send text messages (4 per day) to a study phone during both the three-week baseline and follow-up phases. A memory log ring-binder with a sheet for writing down memory tasks each day was given to participants at the beginning of the baseline period (week 1) and at the beginning of the follow-up period (week 11). Memory log and text time data were analysed for a participant if at least 1 week was completed for both baseline and follow-up phase. Memory log and text time data was combined to create an average memory performance score for the baseline and follow-up phases. This was the average weekly proportion of the total intended memory tasks and text times that were successfully completed. Successful completion of memory log intended tasks was graded depending on if the task was completed (1 point), completed on time (1 point) making a 3-point scale for the memory logs (from 0 to 2). An extra item was added after the trial had started (after three weeks when four participants had been enrolled). This item was added to capture independent remembering; an extra point if the task was completed without prompting from somebody else. This change meant that a 4-point scale (from 0 to 3) was available for participants enrolled after this change was added (*n* = 35, including 16 of the 19 participants who completed the study with enough data for analysis). Follow-up outcome data was gathered by a researcher who did not randomize participants and was blinded to the intervention condition. Details of the memory log and text time scoring method are available in the supplementary materials (doc. 1).

#### Further outcome measures

Further outcome measures captured were participants’ app feedback, technical issues reported by participants, study experience feedback, and notes and observations to capture factors that may influence feasibility.

App feedback questions were asked in the first follow up session which took place six weeks after receiving the app. Participants were asked the following questions: How well do you feel you can use the app?; How useful have you found using it?; How has the app fitted into the rehabilitation you have received from the community treatment centre staff?; Have you had any issues working the app or phone?; Who, if anyone, has helped you use the app?; Do you have any other general feedback about your experiences with the app? Participants were then given questions covering each aspect shown to influence technology use in the Unified Theory of Acceptance and Use of Technology (UTAUT) model; performance expectancy, effort expectancy, attitude toward using technology, social influence, facilitating conditions, self-efficacy and anxiety (Venkatesh et al., 2003). Responses to these questions were transcribed verbatim by the research team and a thematic analysis was completed by two of the researchers to group together similar answers and capture the most common responses by participants. Each of the UTAUT themes consisted of 3 or 4 statements that participants rated on a six-point scale with 1 being completely disagree and 6 being completely agree (full questionnaire is in the supplementary materials doc. 1). An average score for each UTAUT theme could then be calculated to facilitate analysis of the main issues that may influence app use for those given Google Calendar and ApplTree apps.

### Data analysis

Since this was a feasibility trial that aimed to determine recruitment, retention and adherence, no power analysis was used to determine the sample size required. The trial was initially intended to run for one year, recruiting from the collaborating services and the target recruitment (*n* = 50) was based on the referral numbers in the recruiting services and the proportion of service referrals that would meet the study eligibility criteria. Proportions and 95% confidence intervals (CIs) were calculated using the exact binomial test and used to analyse the primary objectives for feasibility. Baseline characteristics, neuropsychological test scores, UTAUT questionnaire and memory performance data were summarized for each randomized treatment group, and reported using counts and percentages. Categorical variables were reported using mean, standard deviation (SD), whereas median, 25th (Q1) and 75th (Q3) percentiles (interquartile range (IQR)) for continuous variables, depending on the distribution of the data. Some of the secondary feasibility objectives were also analysed using descriptive methods; the number of times family members or caregivers helped people fill out the memory log was noted, technical issues with the apps were noted, protocol breaches and attendance and completion of remote and in-person intervention sessions were noted.

For the app feedback, we wished to evaluate the participants’ experiences of the benefits and weaknesses of the app they were given. To do this Structural Coding was used and was applied according to the outline described in Saldana (2011). This involved one researcher (MJ) initially coding the responses from each of the app feedback questions into codes and grouping them into themes under two main thematic headings; perceived usability and perceived usefulness. Perceived usability themes describe the factors that influenced their perception of how well they could use and learn to use the app. Perceived usefulness themes describe the factors that influenced their perception how useful the app was. A second researcher (JE) then checked a portion (20%) of the coded feedback and any disparity in the theme under which the feedback should be coded was resolved during a discussion. The number of comments made by different participants in each sub-theme was calculated to allow interpretation of how important each theme was to the participants overall. This also allowed a comparison between the number and type of comments in each theme for those who received Google Calendar and those who received ApplTree.

#### Power calculation and the minimal clinically important difference

The data from this trial was intended to inform a sample size power calculation for a future RCT through estimation of the variation in the main trial outcome. The main outcome variable in a future RCT would be the difference between baseline and intervention phase memory performance (memory performance will be calculated by combining memory log and text message task scores).

##### Estimating the minimal clinically important difference (MCID)

The research team met with the clinical teams involved in recruitment for this trial to decide upon a MCID. This meeting took place after the trial was complete in October 2021, but before the clinical teams were aware of study results. During the meeting, the clinical teams communicated their expectation that a 25% increase above previous memory performance constitutes a minimal clinically important difference. The example given was that if 50% tasks were remembered by a client using calendars then they would hope to see 75% of tasks be remembered using a memory aid app.

This difference is what we would expect for a comparison between a non-technological aid and a memory aid app. However, we expect that Google Calendar would also have some effect above a non-technological calendar. In our calculations, we assumed that Google Calendar would be half as good as ApplTree. This assumption was made in consultation between the research and clinical teams based on the following reasoning:

- Clinical teams felt it would be worth their time to implement an assistive technology intervention if it led to people remembering twice as many of their tasks than they did before. This was taken as the benchmark for ApplTree to have a clinically important difference.
- However, an app like ApplTree that is developed through clinical research requires time and money to develop and roll-out, so will need to be worth that investment compared to an off-the-shelf app that has been developed and is already demonstrably commercially viable (so will continue being available)
- We know that such off-the-shelf apps like Google Calendar do help people with memory difficulties compared to a non-technological alternative or practice as usual (Jamieson et al., 2014).
- However, we don’t have any data to understand how much better an off-the-shelf app is vs. an app developed by clinical researchers.
- It is not reasonable to expect ApplTree to lead to twice as many tasks being remembered when another technology is the control condition.
- So, it was decided that if Google Calendar sat in between the effectiveness of no-technology/practice as usual (baseline) and ApplTree (best-practice intervention) then that would justify the cost to develop and roll-out ApplTree (or an app with ApplTree’s features) in brain injury services.

Therefore, the minimal clinically important difference between ApplTree and Google Calendar (difference in the average weekly proportion of memory task points scored out of the total possible to score in that week) was set to 0.125 (12.5%). This was used along with the variation of the measure found in the feasibility trial to calculate the effect size to use in a power calculation for a full-scale trial. This variation was calculated as the highest estimation of standard deviation of this measure; the upper limit of the 95% Confidence Interval.

## Results

### Primary objectives

#### Recruitment

Our pre-specified criteria for feasibility was 80% or above recruitment of the planned number of people be recruited into this study (randomized). The recruitment rate calculated at the end of the ApplTree trial was 58% (95% CI = 43.2%, 71.8%; 29 randomized) of the recruitment target of 50.

#### Retention

Our prespecified criteria for feasibility was a retention of 70% or above of randomized participants attending the follow-up sessions until the end of the trial. Our observed retention rate at the end of the trial was 65.5% (95% CI = 45.7%, 82.1%) with 19 of 29 randomized participants attending each of the three follow-up sessions. This proportion was the same for attendance of each of the 1st, 2nd and 3rd follow-up sessions.

#### Adherence

The prespecified criteria for adherence used to judge feasibility were 70% or above of randomized participants using the app they were given until the end of the trial. Use of the app was defined as using the app at least once in each of the three weeks of the follow-up. Of the 29 participants who were given the app intervention and passed the assignments, 14 used the app at least once in each of the three follow-up weeks (48.3%, 95% CI: 29.4%, 67.5%).

### Secondary objectives

#### Details of recruitment, drop-out and non-adherence

##### Recruitment

Over the 14 months where recruitment to the study was open a total of 285 service users were considered by services and 150 met the study criteria. Service staff deemed it appropriate to refer 63 of these 150 people to the research team. Of those referred, 39 consented to being enrolled in the study (61.9% of those referred). Of these, 29 were randomized to receive the intervention (Google Calendar or ApplTree) (46% of those referred).

##### People considered for the study by the service

**Figure 1.**
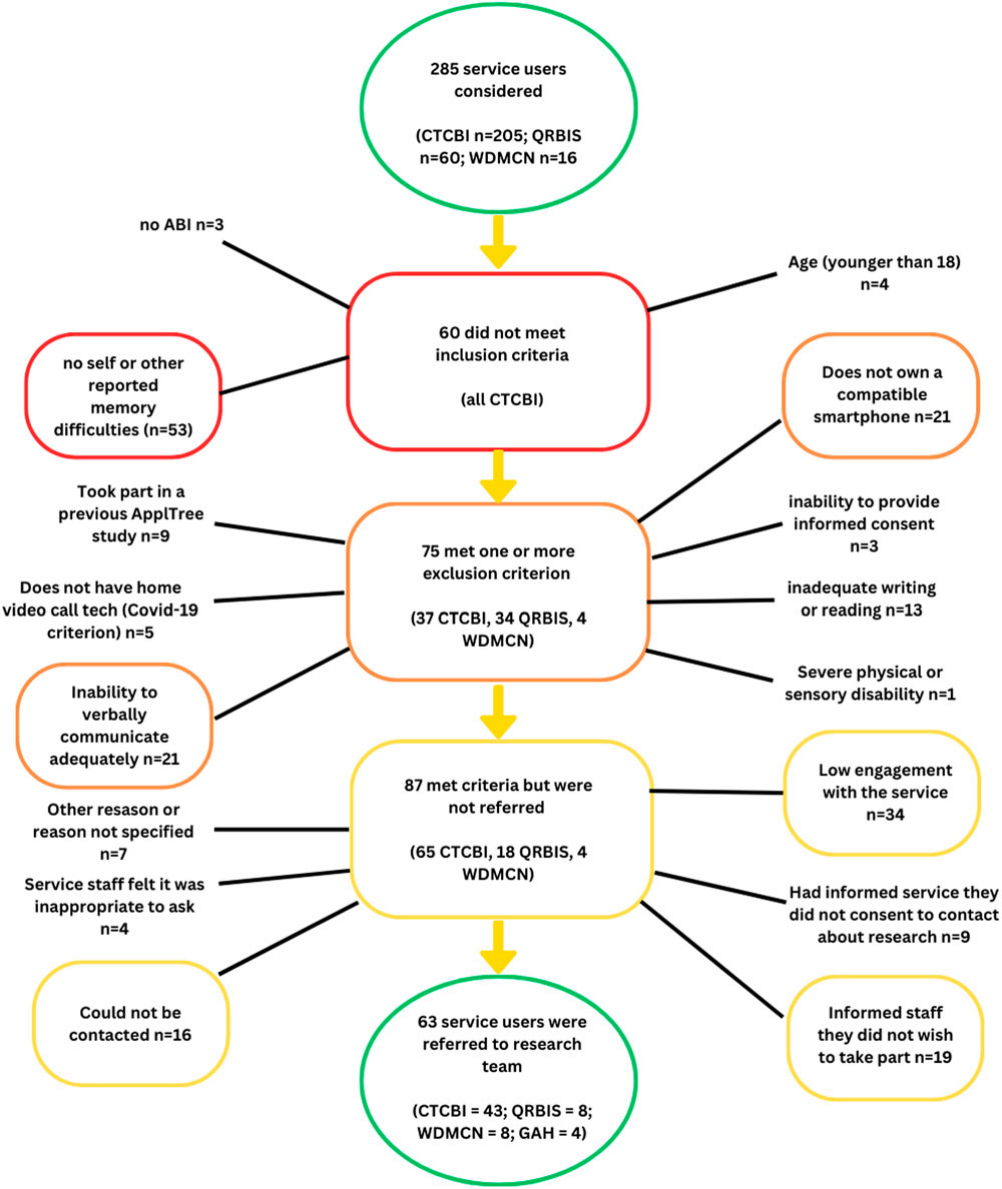
Breakdown of the service users considered but not referred to AppReminders trial.

Figure 1 shows that 285 people (CTCBI *n* = 205; QRBIS *n* = 60; WDMCN *n* = 16; GAH *n* = 4) were considered for the trial over the full recruitment period spanning from July 2019 to April 2021 (inclusive, with 9 month gap between March 2020 and December 2020 due to the Covid-19 pandemic). Of those considered, 222 did not meet criteria or were, for other reasons, not approached by the services to take part.

Of the 285 service users considered over the course of the study, 80 were considered in the 24 weeks following the restart after the stoppage due to the Covid-19 pandemic (CTCBI *n* = 78; QRBIS *n* = 2; WDMCN *n* = 0; GAH *n* = 0). Of the 78 considered following the study restart following the Covid-19 pandemic, 62 did not meet criteria or were, for other reasons not approached by the services to take part.

##### Breakdown of reasons people were not included

Inclusion criteria were checked before exclusion criteria. Most service users who failed to meet the inclusion criteria only failed to meet one inclusion criterion (57 of 60). Of those who met an exclusion criterion, most only met one exclusion criterion (66 of 75). Three service users (all from CTCBI) failed to meet 2 inclusion criteria, 7 (6 from CTCB and 1 from WDMCN) met two exclusion criteria and 2 were noted to meet three or more exclusion criteria (both from CTCBI). Figure 1 shows a breakdown of the number of people failing to meet each inclusion or meeting each exclusion criteria. Each person was only counted once; if there was more than one reason for not including a service user then person was tallied in the first excluding criterion noted by the service.

##### Referrals

**Figure 2.**
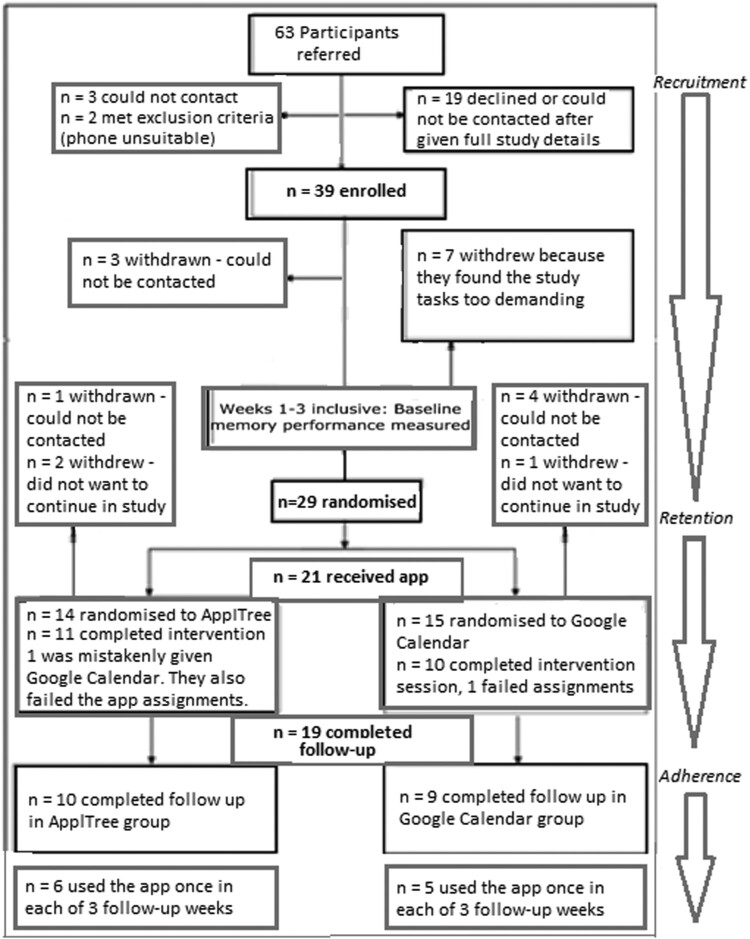
The trial flowchart with enrolment, recruitment, retention and adherence for the 63 referrals to the AppReminders trial.

The remaining 63 participants were referred to, and approached by, the research team: Of those, 24 did not take part. This was because they did not respond to initial contact from the research team (*n* = 3), because they either decided not to participate or could otherwise not be contacted after the researcher went through the details of the study (*n* = 19), or because they were found not to meet the criteria by the research team prior to enrolment (*n* = 1) or during the enrolment session (*n* = 1). Both the participants referred who did not meet criteria did not have a phone that they could use to download the apps. Figure 2 shows the referral, enrolment and recruitment pathway through the study for the 63 participants referred to the study with breakdowns of the numbers of participants at each stage.

##### Retention at each study phase

###### Enrolled participants

The remaining 39 consented to take part in the study. Of these, 29 completed the baseline phase of the study and were randomized (74.4% (95% CI: 57.9%, 87.0%) of the participants enrolled). Table 1 outlines the baseline characteristics of the randomized participants. Ten participants withdrew from the study at baseline. Seven participants withdrew themselves during the three-week baseline phase and three were withdrawn by the research team because they could not be contacted during the baseline phase. The seven who withdrew themselves from the study during the baseline phase said they found it too demanding to do the memory logs, text times and weekly calls. No adverse or serious adverse events were reported for any participants enrolled into the trial.

**Table 1.** Baseline characteristics for the randomized participants.

Variable	Summary Statistic	All Randomized (*N* = 29)	Google Calendar (*N* = 15)	ApplTree (*N* = 14)
Age at baseline (years)	Mean (SD)	47.3 (12.3)	46.9 (12.5)	47.8 (12.5)
Gender	*N* (%) Male *N* (%) Female	10 (34.5%) 19 (65.5%)	5 (33.3%) 10 (66.7%)	5 (35.7%) 9 (64.3%)
Community Brain Injury Service	*N* (%) CTCBI *N* (%) WDMCN *N* (%) BIRT *N* (%) Quarriers	17 (58.6%) 5 (17.2%) 0 (0.0%) 7 (24.1%)	9 (60.0%) 2 (13.3%) 0 (0.0%) 4 (26.7%)	8 (57.1%) 3 (21.4%) 0 (0.0%) 3 (21.4%)
Scottish Index of Multiple Deprivation (SIMD) 2020 Quintile (*n* = 2 data missing; *n* = 14 Google Calendar, *n* = 13 ApplTree)	*N* (%) 1 = most deprived *N* (%) 2 *N* (%) 3 *N* (%) 4 *N* (%) 5 = least deprived	9 (33.3%) 7 (25.9%) 3 (11.1%) 5 (18.5%) 3 (11.1%)	4 (28.6%) 4 (28.6%) 1 (7.1%) 4 (28.6%) 1 (7.1%)	5 (38.5%) 3 (23.1%) 2 (15.4%) 1 (7.7%) 2 (15.4%)
Living Situation	*N* (%) Supported accommodation *N* (%) Living alone *N* (%) Living with family/ partner *N* (%) Living with flatmates/ friends *N* (%) Other	0 (0.0%) 13 (44.8%) 15 (51.7%) 1 (3.4%) 0 (0.0%)	0 (0.0%) 7 (46.7%) 8 (53.3%) 0 (0.0%) 0 (0.0%)	(0.0%) 6 (42.9%) 7 (50.0%) 1 (7.1%) 0 (0.0%)
Time since brain injury, in months	Median [IQR]	24.0 [12.0, 110.0]	24.0 [12.5, 81.0]	30.0 [12.8, 198.5]
Time since index brain injury, in years	Mean (SD) Median [IQR] Min, Max	8.4 (11.9) 2.0 [1.0, 9.2] [0.2, 50.0]	7.0 (9.9) 2.0 [1.0, 6.8] [0.2, 35.0]	9.9 (13.9) 2.5 [1.1, 16.5] [0.6, 50.0]
Aetiology of index brain injury	*N* (%) TBI *N* (%) Other ABI	14 (48.3%) 15 (51.7%)	8 (53.3%) 7 (46.7%)	6 (42.9%) 8 (57.1%)
Currently working or in education	*N* (%) Yes *N* (%) No	8 (27.6%) 21 (72.4%)	4 (26.7%) 11 (73.3%)	4 (28.6%) 10 (71.4%)
Smartphone Type	*N* (%) iOS *N* (%) Android *N* (%) Other	11 (37.9%) 18 (62.1%) 0 (0.0%)	4 (26.7%) 11 (73.3%) 0 (0.0%)	7 (50.0%) 7 (50.0%) 0 (0.0%)
Smartphone use frequency	*N* (%) Very often *N* (%) Often *N* (%) Sometimes *N* (%) Rarely *N* (%) Never	13 (44.8%) 10 (34.5%) 3 (10.3%) 2 (6.9%) 1 (3.4%)	7 (46.7%) 4 (26.7%) 2 (13.3%) 1 (6.7%) 1 (6.7%)	6 (42.9%) 6 (42.9%) 1 (7.1%) 1 (7.1%) 0 (0.0%)
Smartphone calendar use frequency (*n* = 1 data missing; *n* = 14 Google Calendar, *n* = 14 ApplTree)	*N* (%) Very often *N* (%) Often *N* (%) Sometimes *N* (%) Rarely *N* (%) Never	7 (25.0%) 2 (7.1%) 5 (17.9%) 5 (17.9%) 9 (32.1%)	3 (21.4%) 1 (7.1%) 2 (14.3%) 3 (21.4%) 5 (35.7%)	4 (28.6%) 1 (7.1%) 3 (21.4%) 2 (14.3%) 4 (28.6%)
Usefulness of smartphone calendar in helping person to remember (not answered if they never use a smartphone calendar, *n* = 19, *n* = 9 in Google Calendar group, *n* = 10 in ApplTree group)	*N* (%) Vital in helping *N* (%) Often helps *N* (%) Sometimes helps *N* (%) Rarely helps *N* (%) Does not help	8 (42.1%) 7 (36.8%) 2 (10.5%) 1 (5.3%) 1 (5.3%)	3 (33.3%) 3 (33.3%) 1 (11.1%) 1 (11.1%) 1 (11.1%)	5 (50.0%) 4 (40.0%) 1 (10.0%) 0 (0.0%) 0 (0.0%)
Non-electronic calendar use	*N* (%) Yes *N* (%) No	22 (75.9%) 7 (24.1%)	12 (80.0%) 3 (20.0%)	10 (71.4%) 4 (28.6%)
Usefulness of non-electronic calendar in helping person to remember (only answered by those who use non-electronic calendars, *n* = 12 Google Calendar group, *n* = 10 ApplTree group)	*N* (%) Vital in helping *N* (%) Often helps *N* (%) Sometimes helps *N* (%) Rarely helps *N* (%) Does not help	13 (59.1%) 3 (13.6%) 4 (18.2%) 1 (4.5%) 1 (4.5%)	8 (66.7%) 3 (25.0%) 1 (8.3%) 0 (0.0%) 0 (0.0%)	5 (50.0%) 0 (0.0%) 3 (30.0%) 1 (10.0%) 1 (10.0%)

###### Randomized participants

Of the 29 participants who were randomized, 7 left the study before the intervention session could take place. These participants gave enough data initially to be randomized (at least 1 week of baseline data after 3 weeks) and so were randomized as per-protocol. However, they either withdrew before the intervention session (*n* = 3) or could not be contacted to arrange it (*n* = 4). The intervention session was attended by 22 of the randomized participants. Of those, 19 completed at least one week of memory log data during the follow-up phase.

One participant could not complete the intervention session due to a lack of internet connection and lack of expertise connecting to the Google Play store (this was post Covid-19 so was a remote session). Two participants were removed from the study, per protocol, because they failed the app use assessment during the intervention session (both received the Google Calendar app). Of the 19 who passed the intervention session assignment, 16 reached the pass mark on the first of the 5 assignments, 3 reached the pass mark on the second assignment. No participants who passed were required to move to the third of the 5 assignments. All participants who completed the intervention session and passed the assignments provided adequate follow-up data.

Nineteen participants progressed through the full study providing adequate baseline and follow-up data to allow comparison of their memory performance during baseline and follow-up (*n* = 10 were randomized to ApplTree and *n* = 9 were randomized to Google Calendar). Four participants did not provide week 3 of either the baseline or follow-up sessions and so only weeks 1 and 2 of each phase were used in the analysis.

##### Adherence to each app

In the ApplTree group adherence to app use was *n* = 8 (57% of those randomized to the ApplTree group, 80% of those who completed the intervention session), and in the Google Calendar group this was *n* = 6 (40% of those randomized to the Google Calendar group, 67% of those who completed the intervention session). Two participants (11%), one in the ApplTree group and one in the Google Calendar group reported not using the app at all after the intervention session. App use data was gathered from the ApplTree database for all ten participants with ApplTree. Three participants with Google Calendar were happy for app use to be manually checked by the researcher. The other six had the final study session as a phone call due to Covid-19 restrictions and were unable to virtually share their calendar so they all read out the number of reminders in the calendar for each day of the 3 follow-up weeks.

#### To inform the development of a randomized controlled trial: ii) power analysis for full-scale RCT

We aimed to use the feasibility trial data to find out the variation in memory performance and difference in memory performance between baseline and intervention for people in ApplTree and Google Calendar groups. When combined with the primary objective regarding recruitment this gives an indication of how many people should be recruited to adequately power a randomized controlled trial. Table 2 shows the variation in memory performance of those who completed the full trial.

**Table 2.** Change in memory performance for participants who completed the study in both ApplTree and Google Calendar randomized groups.

Variable	Summary Statistic	All Randomized (*N* = 29)	Google Calendar (*N* = 15)	ApplTree (*N* = 14)
Completed follow-up	*N*	19	9	10
Average overall memory performance score – change from baseline (calculated from the sum of the daily text score and memory performance 3-pt scale)	*N* (*N*_missing_) Mean (SD)	19 (0) 0.00 (0.11)	9 (0) −0.02 (0.07)	10 (0) 0.01 (0.13)

Table 2 shows that the variation (SD) of our chosen memory performance outcome score was 0.11. The 95% confidence interval of this SD score is 0.08 to 0.16. We chose to use the highest value as the most conservative choice for inclusion in the power analysis for a future trial (0.16). The MCID that was decided upon following discussion with collaborating clinical teams was 0.125 (a 12.5% difference between ApplTree and Google Calendar with ApplTree improving memory performance).

Effect size (*d*) was calculated by dividing the difference between randomized groups by the highest estimate within 95% confidence interval of SD (change from baseline to intervention):

d=0.125/0.16=0.78

A power analysis was run in G*Power 3.1. Alpha level was set as 0.05, Power to 0.9 and *d *= 0.78*.* This analysis indicated that a full trial would require a sample size of 72 (36 in each group) to be adequately powered to find the effect should it exist.

#### Recruitment requirements for full-scale trial

The figures gathered from the feasibility trial indicate that if we want to end up with 36 people in each group (*n* = 72) we will need to assume the following:

Based on our retention data, the 72 participants who complete the study will be 65.5% of those we randomized. So in the full trial 110 participants would need to be randomized (72/0.655).

Based on our feasibility data, the 110 participants randomized will be 46% of those referred. So 240 people would need to be referred by services (110/0.46).

The 240 people referred to the study (met criteria and referred by services) will be 22.1% of those considered by services. So, 1086 people would need to be considered by services during the course of the trial (240/0.221). In the feasibility trial 63 of 285 people considered for the trial were referred.

##### ApplTree vs. Google Calendar comparison

There was no significant difference between the baseline and post-intervention memory ability score for the combined groups (*n* = 19); 0% (SD = 11%) change in memory performance score 60% (SD = 14%) at baseline and 60% (SD = 14%) at follow-up. For all participants in the ApplTree group (*n* = 10) there was a 1% increase in average weekly memory performance from baseline (SD = 13%); from 55% (SD = 15%) at baseline to 56% (SD = 12%) at follow-up. Those in the Google Calendar group (*n* = 9) had slightly worse memory at follow-up (a decrease of 2% (SD = 7%) from 65% (SD = 13%)) at baseline to 63% (SD = 16%) at follow-up. Calculated using a two-sample unpaired t-test, the estimated mean difference between groups is 0.032 (95% CI: −0.073, 0.137), *p* = .513.

There was a larger difference for the memory log score alone. For all participants (*n* = 19) there was a 7% increase in average weekly memory performance from baseline (SD = 11%); from 86% (SD = 12%) at baseline to 93% (SD = 9%) at follow-up. While the memory log average weekly scores did not increase for the Google Calendar group (*n* = 9) (score went from 93% (SD = 6%) at baseline to 93% (SD = 10%) at follow-up) the score increased by 13% (SD = 8%) for the ApplTree group (*n* = 10; 80%, SD = 13%) at baseline to 93% (SD = 8%) at follow-up. Calculated using a two-sample unpaired t-test, the estimated mean difference between groups with this memory performance score is 0.125 (95% CI: 0.039, 0.21), *p* = .007.

When the altered memory log scoring system that included an item about whether or not people needed to be prompted about each task was used, there was no difference between baseline and follow-up for the combined groups (*n* = 16) in terms of change from baseline to follow-up. There was also no difference for either the Google Calendar (*n* = 9; 0% change, SD = 4%) or ApplTree (*n* = 7; 0% change, SD = 7%) groups. Calculated using a two-sample unpaired t-test, the estimated mean difference between groups with this memory performance score is 0.003 (95% CI: −0.055, 0.062), *p* = .908.

#### Experiences of using the memory aid apps

##### The UTAUT questionnaire

Table 3 shows the Wilcoxon Mann–Whitney analysis results comparing the average scores for each of the UTAUT domains. Two of the eight domains included in the UTAUT were found to have significant differences between the groups. Participants in the ApplTree group (*n* = 10) rated their performance as significantly better than the participants in the Google Calendar group (*n* = 8) (median was 5.7 out of 6 (IQR = 5.1–5.9) for the ApplTree group compared to 4.8 (IQR = 4.2–5.3) for the Google Calendar group) (*p* = .022). Participants in the ApplTree group (*n* = 10) had significantly higher social influence scores than the participants in the Google Calendar group (*n* = 8) (median was 6 out of 6 (IQR = 5.4–6) for the ApplTree group compared to 4.1 (IQR = 2.1–5.1) for the Google Calendar group) (*p* = .015).

**Table 3.** Differences in UTAUT domain scores between ApplTree and Google Calendar groups.

Variable	Summary Statistic	All Randomized (*N* = 29)	Google Calendar (*N* = 15)	ApplTree (*N* = 14)	*p*-value
Received intervention	*N*	21	10	11	–
Performance expectancy domain average score	*N* (*N*_missing_) Median [IQR]	18 (3) 5.3 [4.7, 5.7]	8 (2) 4.8 [4.2, 5.3]	10 (1) 5.7 [5.1, 5.9]	*p* = .022
Effort expectancy domain average score	*N* (*N*_missing_) Median [IQR]	18 (3) 5.8 [5.1, 6.0]	8 (2) 5.4 [4.2, 5.8]	10 (1) 6.0 [5.6, 6.0]	*p* = .162
Attitude towards using technology domain average score	*N* (*N*_missing_) Median [IQR]	18 (3) 5.1 [4.8, 5.7]	8 (2) 4.8 [4.4, 5.0]	10 (1) 5.2 [5.1, 5.7]	*p* = .164
Social Influence domain average score	*N* (*N*_missing_) Median [IQR]	18 (3) 5.2 [3.6, 6.0]	8 (2) 4.1 [2.1, 5.1]	10 (1) 6.0 [5.4, 6.0]	*p* = .015
Facilitating Conditions domain average score	*N* (*N*_missing_) Median [IQR]	18 (3) 5.1 [4.8, 5.9]	8 (2) 5.2 [4.8, 5.6]	10 (1) 5.1 [4.6, 5.8]	*p* = .752
Self-efficacy domain average score	*N* (*N*_missing_) Median [IQR]	18 (3) 6.0 [5.8, 6.0]	8 (2) 5.9 [5.8, 6.0]	10 (1) 6.0 [5.8, 6.0]	*p* = .541
Anxiety domain average score	*N* (*N*_missing_) Median [IQR]	18 (3) 5.8 [5.1, 6.0]	8 (2) 6.0 [5.4, 6.0]	10 (1) 5.5 [5.1, 5.9]	*p* = .376
Behavioural intention to use the system domain average score	*N* (*N*_missing_) Median [IQR]	18 (3) 6.0 [5.7, 6.0]	8 (2) 6.0 [5.9, 6.0]	10 (1) 6.0 [5.4, 6.0]	*p* = .491

##### Participant interviews

Transcripts of interviews where participants were asked about their experiences using the memory aid apps (ApplTree and Google Calendar) were coded using Structural coding and organized in a thematic analysis. A proportion (20%) of the data was double coded by a secondary experimenter. The themes were structured into issues impacting Usability and Usefulness. Central sub-themes for Usability and Usefulness could be split into Facilitators and Barriers.

**Figure 3.**
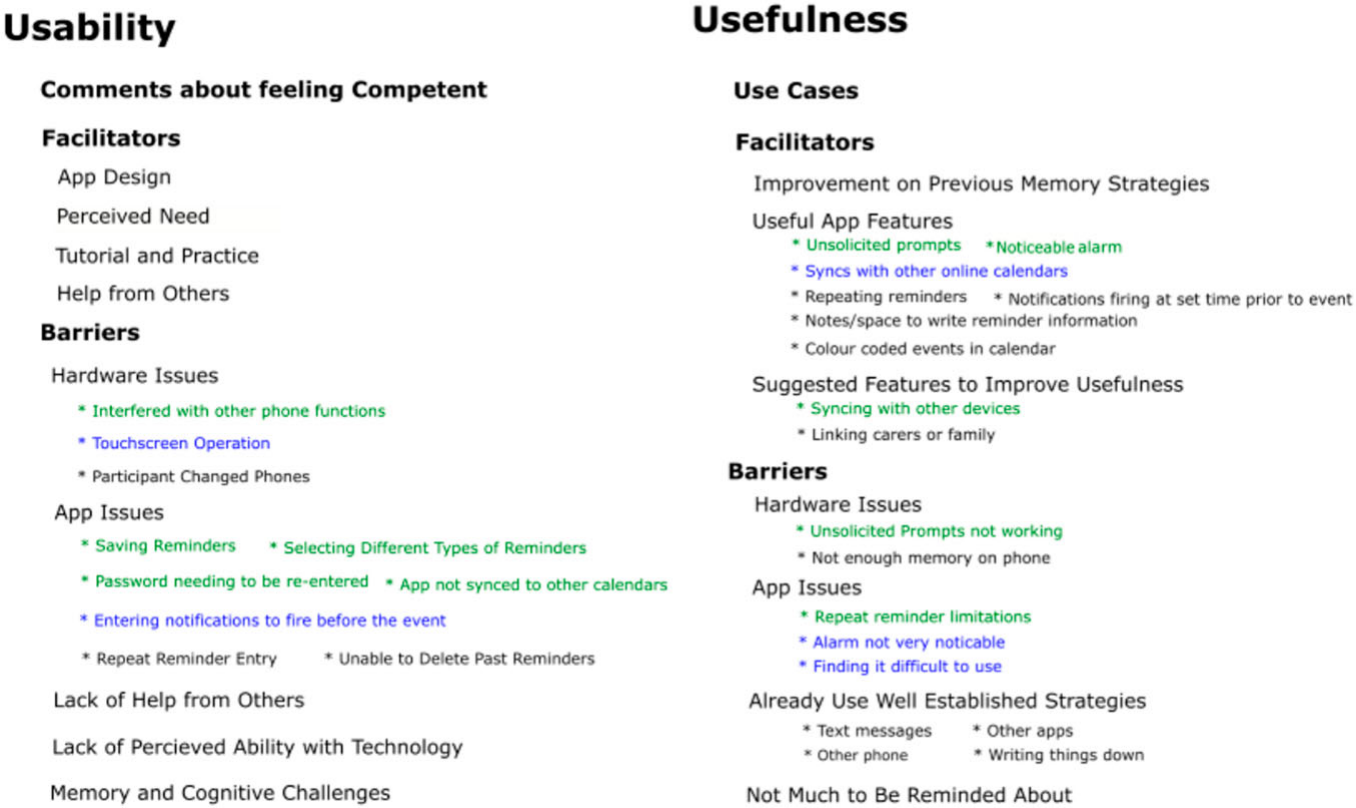
Themes and sub-themes that arose from participants’ feedback after they were given ApplTree or Google Calendar apps.

Facilitators in the Usability themes were *App Design*, *Tutorial and Practice*, *Perceived Need* and *Encouragement or Help from Others*. Barriers to usability were *Hardware Issues*, *App Issues*, *Lack of Perceived Ability with Technology*, *Lack of Help from Others* and *Memory or Cognitive Difficulties*. Facilitators in the Usefulness theme included *App Better than Previous Memory Strategies*, *Useful App Features* and *Suggested Features to Improve Usefulness*. Barriers to usefulness included *App Issues*, *Hardware Issues*, *Previous Use of Well Established Strategies*, and *Not Having Much to be Reminded About*. Other comments in the Usability theme that were not about facilitators and barriers to usability were *Comments About Competency with the App.* Other comments in the Usefulness theme that were not about facilitators and barriers to usefulness were *Use Case Examples*. Figure 3 summarizes the themes and sub-themes for both Usability and Usefulness.

The feedback was split between feedback from participants who received ApplTree and feedback from participants who received Google Calendar. We were especially interested in where there were big differences in the feedback given by the two groups as this would indicate how the experiences using the two apps differ. In figure one the themes that were specific to one app were represented in blue (Google Calendar) and green (ApplTree). The detailed breakdown of the themes below is therefore focused on those themes that had disparities between the two groups.

##### Facilitators and barriers to usability

Four sub-themes of the *Facilitator to Usability* theme had more comments from the ApplTree group than the Google Calendar group. These were *App Design*, *Tutorial and Practice*, *Perceived Need* and *Help or Encouragement from Others*.

*App Design* was mentioned by participants in the ApplTree group who felt the design helped them to use the app. One participant felt he did not require much tuition because the app was designed in a straightforward way;

As I say it’s very intuitive and straight away when (clinician) sat me down to give me a brief overview I thought I really don’t need this in too much depth cos you can see the way it’s been designed that it’s quick and straight forward. (Participant 102)

Participants from both groups discussed the *Tutorial and Practice* as *Facilitators to Usability*. People in the ApplTree group discussed learning to use the app in their own time;

There was a couple of things at first I wasn’t sure of but I soon figured it out. Like to see what these days were (shows researcher events in the calendar screen) but I soon figured out oh just touch it and it brings it all up. So, I mean, that wasn’t explained but it wasn’t very difficult. You just touch on the day and it brings it up and then it takes it away again. (Participant 201)

Another *Facilitator to Usability* was *Perceived Need*. All *Perceived Need* comments came from three participants in the ApplTree group, for example:

This (app) is meant for me. I try not to write things down. If I made a list for shopping I would actually forget the list! Whereas I take this now. I’m still not used to the phone, but when I take that (the phone) out (I can use it when out and about). (Participant 116)

*Help or Encouragement from Others* was another facilitator to usability and this theme had more comments from ApplTree participants than Google Calendar participants in the facilitator of help from others. The only comments in the theme of *Not Receiving Help from Others* – a *Usability Barrier* – were from Google Calendar participants. The quote below highlights the importance of having help from others, even when the person did feel able to use the app by themselves:

I think if I’m right I only stopped it (the ApplTree app) a couple of times to go back just to go over something. But I kinda did pick up on it pretty well I think. I’m sure it was only once of twice I had to stop it. And (the staff member who ran the session) was a great help as well. (Participant 106)

*Usability Barriers* included *Hardware Issues*. One participant in the ApplTree group reported interference with the phone call function and a participant in the Google Calendar group mentioned difficulty working the touchscreen. More common barriers were to do with the app itself. For example, ApplTree participants had issues saving reminders, selecting different types of reminders, had password issues, and found it inconvenient that the app did not sync to other apps. One Google Calendar participant noted issues with adding notifications in the way they wanted. Participants from both groups reported issues with knowing how to delete reminders.

Further issues that impacted usability for both apps included a *Lack of Perceived Ability with Technology* and *Memory and Cognitive Challenges.* Some people did not feel they were good with technology, and this led to a preference for non-technological strategies. Cognitive challenges like memory difficulty could also be a barrier, for example if people forgot to use the app to set a reminder or found the task of putting information into the app challenging. These two themes may have been linked if people felt their cognitive challenges stopped them from being able to learn to use new technology and resulting in a perception that they were unable to use technology.

##### Facilitators and barriers to usefulness

*Useful App Features* was a facilitator to the usefulness of the app that many participants commented on. The unsolicited prompts and having a noticeable alarm were positives noted by people in the ApplTree group. Participants in the Google Calendar group were positive about the app syncing with other calendars. One comment from a participant about ApplTree differentiated it from Google Calendar (an app he had used previously) because of the unsolicited prompt feature. Both apps had positive feedback about the space for notes and colour coded calendar.

One participant discussed the unsolicited prompts as one benefit of the ApplTree app over the Google Calendar app (which he had used prior to the trial):

Because its not identical to Google Calendar – I think its better. As I say its … the frequent reminders prompting you I found the first time round, second time round, I found irritating but then you grew to love it. Cos I’m thinking why are you asking me if I need to set a reminder? Then you reflect on it. I’ve actually remembered a few reminders that I’d forgotten. So it has helped me. (Participant 102)

Another participant said the unsolicited prompts in ApplTree asking him if he needed to set any reminders helped him use the app successfully to prompt him to take seizure medication and reduce his seizures:

Participant: “It’s a good app it keeps sending you reminders if you need to set any reminders.”

Experimenter: Ok. And have you found those useful?

Participant “Yes for my medication. I’ve been on time with my medication.”

Experimenter: And have you been generally setting reminders when you get those prompts? Or do you just use those prompts (for the medication)?

Participant: “Yeah I’m setting it for my medication. See I’ve not been out much cos of this (Covid-19 pandemic lockdown) so … ”

Experimenter: In general how useful have you found using the app since you’ve had it?

Participant: “Very useful. It’s useful for keeping appointments and my medication. So I’ve been taking my medication in time and there’s been less fits. So that’s good haha!” (Participant 302)

Other participants valued the noticeable alarm in ApplTree (a customized alarm designed to go off for 30 s):

Participant: yeah – cos it goes off like, it goes off like an alarm on your phone so I do I find it quite useful.

Experimenter: Are you finding that quite noticeable when it’s going off?

Participant: uhu, sometimes I’m like oh whats that noise! And I’m still like that, what’s that noise?! No, I do – it’s noticeable, it was loud and it vibrates as well. So if it’s in my bag or something like that I can hear it. (Participant 133)

Participants in the Google Calendar group reported liking the sync feature which facilitated switching between personal and work devices:

Better than what I thought. And probably on the plus side it actually syncs with my work calendar. Because that one of my biggest problems of trying to rely on paper. So it’s much better that it syncs with that. Makes life a lot easier. (Participant 109)

*Barriers to Usefulness* included *App Issues*. A specific issue for some ApplTree users was limitations to the repeat reminder functionality; due to issues discovered during development, the number of repeated reminders was limited on iOS phones and individual repeated reminders could not be edited – editing and deleting a repeated reminder changed all reminders in the repeated series. *Hardware Issues* were also reported to impact usefulness for ApplTree; the unsolicited prompts did not work for all participants due to problems with certain phone makes connecting to the ApplTree server.

There were some usefulness issues that impacted ApplTree and Google Calendar participants equally. For example, some participants in both groups felt the apps were not an *Improvement on Previous Memory Strategies,* especially when they *Already Use Well Established Strategies* (e.g., writing things down, using another app or receiving texts from friends of family)*.* Some people also had a feeling that they had *Not Much to be Reminded About.* This was especially the case during the Covid-19 pandemic when there were lockdowns that paused participants’ social lives and daily activities.

#### Neuropsychological test scores

Due to difficulties completing neuropsychological test batteries remotely, many of the neuropsychological tests and sub-tests could not be completed by all participants. Table 4 shows the neuropsychological sub-test scores broken down by intervention group for the tests that were completed by at least 10 participants in each group. These were the tests that could be completed with participants both before and after the covid-19 pandemic. These tests were in person prior to the pandemic and delivered remotely upon the restart of the trial. No large discrepancies can be seen between groups on these measures.

**Table 4.** RBMT verbal memory, prospective memory and orientation plus TEA elevator counting.

Variable	Summary Statistic	Google Calendar (*N* = 15)	ApplTree (*N* = 14)
Verbal memory scaled score	*N* (*N*_missing_) Mean (SD)	11 (4) 9.1 (3.0)	11 (3) 9.0 (2.3)
Verbal memory percentile rank	*N* (*N*_missing_) Mean (SD)	11 (4) 41.5 (31.3)	11 (3) 41.6 (24.5)
Prospective memory scaled score	*N* (*N*_missing_) Median [IQR]	11 (4) 8.2 [5.5, 9.8]	11 (3) 7.0 [5.5, 8.8]
Prospective memory percentile rank	*N* (*N*_missing_) Median [IQR]	11 (4) 25.0 [7.0, 50.0]	11 (3) 16.0 [7.0, 37.5]
Orientation to date/time scaled score	*N* (*N*_missing_) Median [IQR]	11 (4) 8.0 [4.5, 11.0]	11 (3) 8.0 [6.0, 11.0]
Orientation to date/time percentile rank	*N* (*N*_missing_) Median [IQR]	11 (4) 25.0 [3.6, 63.0]	11 (3) 25.0 [9.0, 63.0]
Elevator Counting percentile	*N* (*N*_missing_) Median [IQR]	10 (5) 6.5 [5.2, 7.0]	11 (3) 6.0 [5.0, 6.5]

Overall combined results indicate average verbal memory ability (mean percentile rank of 41.6 (SD = 24.5) for the ApplTree group and 41.5 (SD = 31.3) for the Google Calendar group). Slightly below average prospective memory was observed for the participants (median percentile rank of 16.0% (IQR = [7.0, 37.5])) in the ApplTree group and 25.0% (IQR = [7.0, 50.0]) in the Google Calendar group). The scores for orientation to time and date were similar (median percentile rank 25.0 (IQR = [3.6, 63.0]) for the Google Calendar group and 25.0 (IQR = [9.0, 63.0]) for the ApplTree group. Elevator counting is a measure of sustained attention. The median score was 6.5 (out of 7 (IQR = [5.2, 7.0])) for the Google Calendar group and 6.0 (IQR = [5.0, 6.5]) for the ApplTree group. As the normative sample scores were at ceiling for this measure, a score of 6 indicates possible difficulties and below 6 indicates impaired sustained attention.

## Discussion

The results of this pilot feasibility trial can directly inform the development of a full randomized controlled trial comparing a technology to support memory for people with ABI to a widely used and commercially available alternative. For this RCT, where a minimal clinically significant effect size of *d *= 0.78 was assumed, 240 participants would need to be referred via services to assure the recruitment, retention and adherence rates required to have 72 participants fully complete the trial. This would take 4 years to complete assuming the same number of services were involved and assuming the same referral rate that was observed in this pilot feasibility trial. An RCT scaled up to involve services with double the number of referrals could be completed in half this time.

### Recruitment challenges

Recruitment was lower than the prespecified target for feasibility (58% compared to 80%). Retention was also below our prespecified target; 65.5% compared to 70% of people staying in the study until the end after being randomized to condition. The challenges of recruiting people who are receiving community care for ABI are highlighted in this trial. It is notable that although 150 service users (53% of those considered by services) met the study criteria, only 63 were referred. This was largely because of low engagement with services (which meant the service made a judgement not to ask the person), because the service could not get in touch with them and because the service users had already informed the staff they did not want to take part in any research. During the trial, existing challenges were exacerbated by the lockdown following the Covid-19 pandemic which impacted the usual running of community rehabilitation services. For example, recruitment via two services was reduced or stopped post-pandemic due to challenges in these service that meant the services were taking on fewer new patients than they expected to. The CTCBI service that accounted for most of the participants in this trial also reported substantially fewer referrals in the year following March 2020.

### Intervention feasibility

The adherence to use of the app was lower than our prespecified target of 70%. Instead, 48.3% randomized participants used the app in each of the three follow-up weeks. This is a low estimate of adherence in practice because only 19 of the 29 randomized participants attended the intervention session, passed the assignments and received the app. The proportion of those who passed the intervention session app assignments who used the app in each of the three follow-up weeks was 73.7% (14 of 19).

There are positive signs that the intervention is feasible to introduce in clinical practice. The intervention session was very brief (45 min to 1 h). However, the majority (19 of 21) of participants who stayed in the study through the baseline phase and attended the intervention session, were capable of learning to use the app in a short session with a video tutorial and app use practice session. This is consistent with recent findings in a pilot RCT comparing different training methods for the Cozi app for people receiving ABI rehabilitation (Ramirez-Hernandez et al., 2021). This study found that participants were able to use the app after only a single training session. However, the authors do conclude that some of the benefits of training methods that utilize errorless learning or metacognitive strategies may be more pronounced with training over a longer period with more sessions.

Another aspect relevant to the feasibility of the app intervention in clinical practice is whether the session needs to take place in-person or if a remote session is feasible. In the trial, 14 participants had in-person sessions with a service staff member before the move to remote sessions and 8 had calls or videocalls with the researcher after the study was made remote. One participant out of the eight who remotely attended the intervention session was unable to complete the session. In this case, it was unclear if he had a phone able to download apps or if he did not have internet connection. It was therefore difficult to tell if a face-to-face session would have helped overcome technical issues and allow the app to be downloaded. The other seven were able to download the app, watch the video and passed the assignments. In comparison, all 13 participants attending in person were able to get the app on their phone and two failed the assignments. While there is only a small amount of data, there is no evidence from this trial that this intervention is less feasible when delivered remotely.

A single, short session that could be in-person or remote is likely to be easier to implement in services than training programmes with multiple sessions that needs to be in-person. However, a lot of participants left the trial before attending to the intervention session, mostly because they found the memory logs and text times to be too much to do. It is possible that competing life demands made meeting the trial demands unfeasible. Therefore, it is possible that the participants who attended the intervention session were more motivated to continue to take part, had fewer competing demands, or had a higher level or functioning than average for service users receiving rehabilitation in the services. A single in-person or remote session may be sufficient for these participants to learn how to use the app, but other service users may need more training sessions and require sessions to be in-person.

### Efficacy data

The trial was not powered to assess the differences between the two apps between baseline and follow-up. However, it is noteworthy that there was no difference observed between baseline and follow-up memory performance for the combined sample. The 12.5% minimal clinically important difference (MCID) level (that was decided in consultation with collaborating clinical services) was included within the 95% confidence interval of this point estimate (95% CI for this outcome was between a 7.3% improvement with Google Calendar to a 13.7% improvement with ApplTree). The largest difference was for the ApplTree group when only the original un-modified memory log scale was used as the memory performance measure without adding text times (a 13% increase). This increase in memory ability compared to Google Calendar is similar to the 12.5% MCID. A full trial is necessary to find out if the difference between the apps is at the MCID level. Methodological issues that may influence the findings are discussed in the section below.

### Methodological considerations

#### Measuring memory ability

During this trial, we attempted to measure prospective memory in a robust way to measure the impact of the experimental and control interventions. However, this is difficult to do because the introduction of a memory log can, prior to the study intervention, aid people with organizing and completing daily tasks. Physical memory log folders paired with the instruction to check the memory log daily (to ensure it is filled out accurately) and weekly calls from a researcher to review the log may have been highly motivating for participants. Indeed, many participants reported that they felt the memory log had a positive effect on their memory. The baseline memory performance observed in the feasibility trial was very high; higher than would be expected for a sample of people reporting everyday memory difficulties. For example, when using the memory log scale that took into account only whether the task was completed or not and whether it was on time or late, participants got a weekly average of 86%. A high baseline memory score creates a challenge in this research as an app will not be able to help improve a good level of prospective memory. If the memory logs mean that some participants perform at or close to ceiling in their memory performance at baseline; the difference between this baseline and follow-up (and the difference between improvement from baseline between two interventions) will be inevitably small.

There was a smaller difference between Google Calendar and ApplTree when the text time data was added to the memory logs than when memory logs analysed alone. This may be a coincidence, or it may be that text times were measuring memory more reliably than the memory logs which were subjective and relied on self-report and report from the experimenters. Alternatively, field notes and descriptive analysis of the data indicate that the text time measure was not capturing memory improvement that could be brought about by the introduction of a reminder app. The text time task involved the participants sending texts at 4 randomly allocated times during each day of the baseline and follow-up phases. This information could have been entered into one of the apps though participants were given no specific instruction to do this. Getting full or high marks on this task was quite demanding and it is possible that it became a measure of the extent to which participants were engaged in the trial rather than the extent to which their memory was supported. Many of the participants who completed the trial received very high scores during the baseline phase which were difficult to surpass in the follow-up. Other participants were less engaged with this task and so may have received very low scores during baseline and low scores during follow-up when they remained less engaged with this part of the study.

There are many aspects of everyday functioning that memory aids can help with beyond the completion of intended tasks. For example, it may be that use of a memory aid could improve confidence and/or increase the amount that the person organizes or feels they can take on. The perceptions of family members or carers about the person’s functioning may be different before and after the use of a memory aid; maybe they need to prompt the person less, or see them as more motivated. Measures that include feedback from significant others or the person’s confidence in managing their cognitive difficulties could be used in future research to capture these effects.

#### Deciding on a minimal clinically important effect size

A minimal clinically important effect size was developed in collaboration with the services involved in this study. It was decided that 12.5% more tasks remembered with one memory app intervention over the number that would be remembered with another would be deemed clinically important. This informed our estimation of the necessary recruitment, timeline and scale of a randomized controlled trial that would be sufficiently powered to find this difference should it exist.

The minimal clinically significant difference is concrete, and it is easy to understand why it would be deemed as important to service users and clinicians. However, the resulting effect size of an intervention that improves memory by the minimal clinically important amount varies depending on how memory performance is calculated. In this study, we take difference between the proportion of successfully completed memory tasks in baseline and post-intervention as the outcome variable. In this case, the difference any number of extra tasks remembered each week makes depends on the number of tasks the person intended to complete each week. However, not all memory tasks are equally important; forgetting an appointment or important social event can have a big impact, while forgetting a household task may be far less important. Therefore, an alternative memory measure might be calculating the absolute number of tasks that were forgotten in a day or week, instead of the proportion of intended tasks remembered or forgotten. If the outcome variable is the difference in the absolute number of tasks forgotten each week then an app that helps them remember one or two extra tasks may show a big effect compared to the same memory performance calculated as the proportion of the total tasks remembered or forgotten. It is not clear which method is best because those few timely reminders could be very important to somebody, or hardly noticeable, depending on the person’s situation and memory tasks. Since the size of the effect influences how many participants need to be recruited into a full-scale randomized controlled trial, it is important to recognize the impact that measurement method makes even when the definition of a minimal clinically significant difference remains the same.

### User experience data

Two of the UTAUT domains had significantly higher scores for the participants in the ApplTree group than those in the Google Calendar group. These were “Performance Expectancy” and “Social Influence.” Performance expectancy measures the extent to which users feel they can use the app for its intended purpose. It is possible that the reduced complexity of the ApplTree design with a decision tree design to breakdown the information needed for different types of reminders and the narrow-deep interface which requires the user to enter only one piece of information at a time might account for this difference between the groups. This is in line with previous findings comparing a narrow-deep user interface design to a broad-shallow interface similar to Google Calendar. Participants with ABI (*n* = 32) made fewer errors when setting example reminders with the narrow-deep UI compared to the broad-shallow UI (Jamieson, Lennon, et al., 2022). It is also a possibility that participants in the ApplTree group knew the app was designed by the experimenters (this information was not communicated to the participants in the protocol or study session scripts). It is also likely that most participants in the Google Calendar group would have realized that the researchers had no role in developing Google Calendar. Therefore, it is possible that participants rated ApplTree more favourably than Google Calendar due to observer-expectancy bias.

Participants in the ApplTree group had significantly higher social influence scores indicating that important people in their lives were encouraging and helped them with the ApplTree app, more so than the important people of the participants in the Google Calendar group. This may be because those in the ApplTree group happened to have people who encouraged and helped them with the app while those in the Google Calendar group did not. It may also be the case that family and caregivers found the ApplTree app easier to use themselves and so were more likely to help and offer encouragement. Lack of perceived ability with technology has been shown to contribute to carer hesitancy and clinical decision making when helping people use assistive technology (Jamieson et al., 2020; Taylor-Goh, 2015). Another possibility is that the participants in the ApplTree group were happier to ask for help than those in the Google Calendar group. Future research could consider family and caregiver perspectives and investigate their involvement in use of memory aid technology in rehabilitation.

Participant feedback highlights that the design features implemented in ApplTree have potential to improve the uptake and utility of reminding apps. Themes were broken down into facilitators and barriers to usability (the perceived and actual ability of the person to use the app) and facilitators and barriers to usefulness (defined as how useful the app was at supporting memory). There were some notable differences between the ApplTree and Google Calendar groups.

#### Facilitators and barriers to usability

ApplTree has issues Google Calendar does not have in integrated systems, for example logging in and out and syncing to other apps. The apps had differing software issues related to their user interface style; ApplTree received positive app design feedback that Google Calendar did not get, but difficulties with narrow-deep design included not seeing where to save reminders (as you had to get to last page) and not knowing which type of task to select. ApplTree users also gave comments about learning in their own time that were not made by Google Calendar participants, perhaps indicating that ApplTree UI facilitated them to work it out by trial and error.

Google Calendar participants reported issues with missing notification setting, touchscreen operation and deleting reminders which may be down to the user interface style where all information is presented on a small number of screens. It may be easier to miss things. All issues with memory or cognitive difficulties related to usability were reported by Google Calendar participants possibly indicating that Google Calendar was challenging to use for people with more cognitive difficulties. This may be because 2 people who failed the app assignments were in the Google Calendar group and 2 of the 4 pieces of feedback in this theme came from observations with these participants. It is unclear if they would have passed the assignments and been able to use the app had they been given ApplTree instead.

#### Facilitators and barriers to usefulness

Unsolicited prompting and having a noticeable alarm are features that ApplTree users were very positive about and this links to past research. Having an app sync to other apps is very useful if people are already using apps prior to an app intervention and this could inform what app is chosen in clinical practice. Established apps like Google Calendar are also less likely to have software issues or issues when linking to different types of phones as these issues are dealt with as standard by the companies that provide them.

### Neuropsychological tests

It was initially hoped that secondary analyses could be performed to investigate the link between neuropsychological test scores and app effectiveness. It was subsequently decided that these scores could only be used to provide a detailed description of the cognitive profile of the participants. This decision was made because there were a small number of participants in the trial and because of the reduced number of neuropsychological sub-tests that could be run for all participants before and after the Covid-19 lockdown. Further research that recruited a larger number of participants who all completed the same neuropsychological tests would be better placed to explore the link between cognitive profile and the efficacy of app-based memory interventions.

### Future research

The trial results have indicated some alterations that could be made to future trials. The fact that the study feasibility did not reduce when Covid-19 resulted in an enforced move to remote study sessions is positive. A flexible approach to online or in-person sessions may make it easier for participants to fit the trial around other commitments and reduce costs associated with travelling to and from study sessions. Many participants who enrolled left the study before the end and so a future trial could reduce the amount participants needed to do (e.g., reducing or removing the text-times task or involving a significant other to do some or all of the memory logging).

There was positive feedback amongst participants who received ApplTree about the features specifically designed to help people with ABI get the most out of these technologies. However, this did not translate into differences in memory aid ability using the memory logs. The trial has informed considerations around how we should go about measuring memory ability and the measure the impact that memory aid technology has in ABI rehabilitation. During this study it became clear that having a measure of whether somebody independently remembered something is important, as well as noting if they did the task or not. Future research could consider broader measures of cognitive functioning and feedback from family members to capture the different ways that technology can support people. Memory aid technology is also intended as a long-term compensatory strategy. Therefore, future research could consider longer term follow-up to see what factors and app features lead to uptake and longer term engagement. This is consistent with a recent recommendation following a systematic review of the literature in memory aid technology in brain injury rehabilitation (Ownsworth et al., 2023).

### Limitations

We were not able to meet our target sample size of 50 participants. Documenting the number of participants we were able to recruit is informative about the feasibility of a full-scale trial. This feasibility study paints a realistic picture of the rates of referral, recruitment and study adherence likely when recruiting people through community-based ABI treatment centres. The trial also allowed the piloting of the outcome measures that could be used and this can inform future trials. The small sample size does reduce the confidence in any further analyses such as the descriptive analysis of the difference between baseline and follow-up memory performance and the difference between the two apps in supporting memory. The quantitative usability assessment using the UTAUT would also have given more reliable results if there were more participants who had received the app and completed this questionnaire.

There were potential risks of bias in this trial that are relevant to a future RCT. For example, there may have been referral bias because a high proportion of people considered by the clinical teams to meet the study criteria were not referred due to other reasons. Many (*n* = 34) of these non-referrals were also due to low engagement with the services. In these instances, service staff did not feel it was appropriate to refer a service user to the study when they had not engaged with the service during their rehabilitation. In a small number of cases staff felt it would be inappropriate to ask the person (*n* = 4), or they did not approach them for another unspecified reason (*n* = 7). It is important in clinical studies that services can use their best judgement when approaching potential participants. It is possible that these subjective decisions taken by the clinical team during the referral process may have led to referral bias so that only more engaged participants ended up taking part in the study. Understanding that there is a gap between the number of people who meet the study criteria (in this case, *n* = 150) and the number who are eventually recruited (*n* = 63) is important when thinking about how many people may need to be considered for a future trial, and when creating study criteria and study protocols to ensure people are only approached when it is appropriate. Future work could also consider how to best involve people in research, who may wish to be involved, but have not engaged well with rehabilitation services.

Another risk of bias was from researchers scoring the follow-up memory logs being unblinded to condition. We did find that it was feasible to blind one researcher who then entered the follow-up memory ability data. This researcher was only unblinded once and this can reduce the risk of bias when making decisions about what scores to give participants in the memory logs (e.g., when should a memory task they did not complete be removed because the task was not completed due to reasons outside of their control). Another risk of bias was that participants were not formally blinded to the purpose of the experiment. It is possible that some participants realized that ApplTree had been created by the researchers and that Google Calendar was a commercially available app. This may have led some participants to answer questions positively about ApplTree and for others to not worry about being overly positive about Google Calendar. To reduce this potential an exclusion criterion was added to exclude any participants in previous research with ApplTree from taking part in the trial. However, the two apps were mentioned to participants in the information leaflet prior to consenting to the study. As was necessary to meet ethical standards, participants were told about their information that would be available to the researchers via the ApplTree server if they were to receive that app. In a future trial it might be better to acquire further consent for researchers to access server information only after the participants have been randomized to ApplTree. This could reduce the chances of participants realizing that the ApplTree app was developed by the research team. The coding for the thematic analysis was conducted by the researchers who designed and delivered the intervention meaning there was also the potential for bias in this research in both the questions asked and because of the subjective nature of the analysis. Consensus coding was completed for 20% of the qualitative data from a researcher not directly involved in the intervention delivery (JE) to help reduce the risk of this bias in the analysis.

The Covid-19 pandemic brought challenges to the researchers and participants and resulted in some participants taking part in all aspects of the study remotely. The fact that this trial spans two very different circumstances for services and service users may make the results less generalizable to a future trial. The move to make the study remote also reduced the number of neuropsychological tests that could be completed with all the participants in the study and meant that tests that were designed to be given in person were given remotely. The move to a remote study including remote intervention delivery did allow the trial to inform the feasibility of remote delivery in the future. The remote intervention sessions were generally successful, and this indicates that a remote intervention may be feasible for community brain injury service users. Furthermore, it would be feasible for a future trial to have more remote sessions than we had originally planned for; especially when gathering memory data and getting usability feedback. Sessions gathering this information were completed just as well before and after the move to make the study remote. This could make future research more flexible in circumstances where face-to-face sessions is difficult, for example when working across large distances.

## Conclusions

The features developed and researched in the ApplTree app positively impacted usability and usefulness for people with memory difficulties after ABI. A large-scale clinical trial is needed to evaluate any improvement to memory ability that is created by using an app that has these features compared to a commercially available and commonly used app that does not have these features. Recruitment, retention and adherence were below our prespecified targets indicating that this trial would be a challenge to complete unless scaled-up. The information gathered in this pilot feasibility trial suggests that a full-scale trial would require 72 participants to fully complete the study and that this trial would take four years to run at the current scale (or two years with an increase in services involved that doubles the number of referrals). The short app intervention given to participants in this trial is feasible to implement in practice.

## Supplementary Material

Supplimentary_materials_doc_2.docx

supplimentary_materials_doc_1.docx
