# Supplementary material for: AppReminders – a pilot feasibility randomized controlled trial of a memory aid app for people with acquired brain injury
Source: Neuropsychol Rehabil. 2023 Jun 13;34(4):535–71. doi: 10.1080/09602011.2023.2220969 (PMC11166046; doi:10.1080/09602011.2023.2220969)
Supplement: Supplimentary_materials_doc_2.docx [file PNRH_A_2220969_SM7178.docx]

# App use assignments

There were five assignment sheets in total, each sheet had three reminders. The three reminders on each sheet asked the participants to set three related reminders (e.g. entering charity shop shifts, then later adding a note to the reminder for one of the shifts). Each aspect of these reminders (e.g. title, date, time, repetition) was scored by the clinician or researcher and each sheet had a set pass mark. The sheets were numbered 1-5 and given out in a set order so all participant received the same sheet first. Participants were only given another assignment sheet if they failed one sheet (e.g. if sheet one was failed then the participant would be given sheet two). A time limit of 10 minutes was set for each sheet and the sheet was scored after the 10 minutes even if the participant had not set all the reminders. Participants passed the assignment if they completed one sheet at or above the pass mark and did not need help with at least one of the three reminders on the sheet. Participants failed the assignments if they tried all the sheets without reaching the pass mark, or if the clinician or researcher judged that they were not able to independently set the reminders (this was to avoid participants going through all 5 sheets when it was clear they were not able to use the app).

Assignment 1

You have volunteered in a charity shop this coming Wednesday and the next two weeks from 10am to 4pm.

You need one hour to get ready and travel to the shop.

You need to bring the shop keys with you.

Set the following Appointment:

Title: Charity shop shifts

Date/when**: This coming Wednesday**

Time: 10am

Duration: 6 hours

Repeats?: Yes, Wed + two weeks

Notification: One hour before

Notes or add description: bring keys

Assignment 2

Your friend Jim has organised a lunch at a Café **Friday next week** at 3pm. You need to be reminded 30 mins before to get there on time.

Set the following Social event:

Title: Lunch with Jim

Location: Café

Date/when: **Friday next week**

Time: 3pm

Repeats?: Does not repeat

Notification: 30 mins before

Assignment 3

You want to buy a card for Jim on your lunch break **this Wednesday** at 1pm

Set the following Shopping event:

Title/Where will you shop: Charity shop to buy card

Date/when: **This coming** **Wednesday**

Time: 1pm

Repeats?: Does not repeat

Notification: At time of event

Shopping list/note: Card for Jim

| **Assignment 1** | Correct? | Any notes about mistake, if made | Help needed? |
| --- | --- | --- | --- |
| Name (Charity shop shifts or similar) |  |  |  |
| Date (as asked) |  |  |  |
| Time (10am) |  |  |  |
| Duration (6 hours) |  |  |  |
| Repetition (Wednesday) |  |  |  |
| Repetition (for 3 weeks or + 2 weeks) |  |  |  |
| Notification (1 hour before) |  |  |  |
| Notes (bring keys) |  |  |  |

Sub total = /8

| **Assignment 2** | Correct? | Any notes about mistake, if made | Help needed? |
| --- | --- | --- | --- |
| Name (lunch with Jim or similar) |  |  |  |
| Location (Café or similar) |  |  |  |
| Date/when (as asked) |  |  |  |
| Time (3pm) |  |  |  |
| Repeats (No repetition) |  |  |  |
| Notification (30 mins before) |  |  |  |

Sub total = /6

| **Assignment 3** | Correct? | Any notes about mistake, if made | Help needed? |
| --- | --- | --- | --- |
| Name (charity shop to buy card or similar) |  |  |  |
| Date (as asked) |  |  |  |
| Time (1pm) |  |  |  |
| Repetition (none) |  |  |  |
| Notification (at time of event) |  |  |  |
| Shopping list/note (Card for Jim or similar) |  |  |  |

Sub total = /6

**Total =** /20 Pass mark = 14

Assignment 1

You have been prescribed a two week course of Antibiotics.

Starting **tomorrow**, you need to take these at 12.30pm and 5.30pm before food.

Set the following Medication event:

Title: Antibiotics medication

Date/when: **starting tomorrow**

Repeats?: every day for 2 weeks

Time(s): 12.30pm and 5.30pm

*(with Google Calendar you may need to set two separate events)*

Notification: At time of event

Assignment 2

Your friend Sarah has organised a meal at an Italian bistro **this coming Wednesday** at 5pm.

It will take you 1 hour to get ready & travel there.

Set the following Social Event:

Title: Meal with Sarah

Location: Italian bistro

Date/when: **This coming Wednesday**

Time(s): 5pm

Repeats?: Does not repeat

Notification: One hour before

Assignment 3

You’ll need to take your antibiotic medication before your meal with Sarah on **this coming Wednesday.**

Set the following Other reminder:

Title: Take antibiotics before meal

Date/when: **This coming Wednesday**

Time: 5pm

Repeats?: Does not repeat

Notification: At time of event

| **Assignment 1** | Correct? | Any notes about mistake, if made | Help needed? |
| --- | --- | --- | --- |
| Name (Antibiotics medication) |  |  |  |
| Date/when (starting tomorrow) |  |  |  |
| Repetition (every day) |  |  |  |
| Repetition (for 2 weeks) |  |  |  |
| Time (12.30pm) |  |  |  |
| Second Time (5.30pm)  (additional reminder should be set in Google Calendar) |  |  |  |
| Notification (At time of event) |  |  |  |

Sub total =  **/7**

| **Assignment 2** | Correct? | Any notes about mistake, if made | Help needed? |
| --- | --- | --- | --- |
| Name (meal with Sarah or similar) |  |  |  |
| Location (Italian bistro) |  |  |  |
| Date/when (as asked) |  |  |  |
| Repetition (none) |  |  |  |
| Time (5pm) |  |  |  |
| Notification (1 hour before) |  |  |  |

Sub total = **/6**

| **Assignment 3** | Correct? | Any notes about mistake, if made | Help needed? |
| --- | --- | --- | --- |
| Name (take antibiotic before meal or similar) |  |  |  |
| Date (as asked) |  |  |  |
| Time (5pm) |  |  |  |
| Repetition (none) |  |  |  |
| Notification (at time of event) |  |  |  |

Sub total = **/5**

**Total =** /18 Pass mark = 13

Assignment 1

You are going on a day trip to a safari park.

This trip will take place on **the last day of next month** and begins at 10am.

It takes 30 minutes to get from your house to where the bus will pick you up.

Set the following Social Event:

Title: Day Trip

Location: Safari park

Date/when: **the last day of next month**

Time: 10am

Repeats?: does not repeat

Notification: 30 mins before

Assignment 2

You will need to pay for the trip at regular group meetings over 3 weeks.

Set a reminder to bring £5 to the meeting on Wednesdays from 2pm-3pm for the next 3 weeks beginning **this coming Wednesday**

Set the notification for 1 hour before.

Set the following Other event:

Title: Group meeting

Date/when: **This coming Wednesday**

Time: 2pm

Repeats?: This day, this week + 2 more weeks

Notification: One hour before

Notes/Description: Bring £5

Assignment 3

You’ll need to pack a lunch the evening before the Safari park trip.

Set a reminder to do this for 7pm on **the day before your trip**.

Set the following Other event:

Title: Prepare lunch for trip

Date/when: **the day before your trip**

Time: 7pm

Repeats?: Does not repeat

Notification: At time of event

| **Assignment 1** | Correct? | Any notes about mistake, if made | Help needed? |
| --- | --- | --- | --- |
| Name (day trip or similar) |  |  |  |
| Location (safari park) |  |  |  |
| Date (as asked) |  |  |  |
| Time (10am) |  |  |  |
| Repetition (none) |  |  |  |
| Notification (30 mins before) |  |  |  |

Sub total = /6

| **Assignment 2** | Correct? | Any notes about mistake, if made | Help needed? |
| --- | --- | --- | --- |
| Name (Group meeting) |  |  |  |
| Date/when (as asked) |  |  |  |
| Time (2pm) |  |  |  |
| Repeats (Wednesday) |  |  |  |
| Repeats (3 weeks or + 2 weeks) |  |  |  |
| Notification (1 hour before) |  |  |  |
| Notes/added description (bring £5) |  |  |  |

Sub total = /7

| **Assignment 3** | Correct? | Any notes about mistake, if made | Help needed? |
| --- | --- | --- | --- |
| Name (Prepare lunch or similar) |  |  |  |
| Date (as asked) |  |  |  |
| Time (7pm) |  |  |  |
| Repetition (none) |  |  |  |
| Notification (at time of event) |  |  |  |

Sub total = /5

**Total =** /18 Pass mark = 13

Assignment 1

You need to take Aspirin every day at 12.30pm and 6pm after food.

You need to take this medication for the next 2 weeks.

Set the following Medication event:

Title: Aspirin

Date/when: **Every day of the week from tomorrow**

Repeats?: This week + 1 extra week

Time: 12.30pm and 6pm

*(with Google Calendar you may need to set two separate events)*

Assignment 2

It is your friend Jack’s birthday tomorrow.

You are attending a party starting at a local café at 12.30pm **tomorrow**

You need to be reminded 30 mins before to get there on time.

Set the following Social event:

Title: Jack’s birthday

Location: Café

Date/when: **tomorrow**

Time: 12.30pm

Repeats?: Does not repeat

Notification: 30 mins before

Assignment 3

You have made an appointment to see your GP **this coming Friday** between 2-2.30pm.

You will need to be reminded 1 hour before the event.

You want to remember to mention your repeat prescription.

Set the following Appointment event:

Title: GP appointment

Date/when: **This coming Friday**

Time: 2pm

Duration: 30 mins

Repeats?: does not repeat

Notification: 1 hour before

Notes/Add description: Mention repeat prescription

| **Assignment 1** | Correct? | Any notes about mistake, if made | Help needed? |
| --- | --- | --- | --- |
| Name (aspirin) |  |  |  |
| Date (as asked) |  |  |  |
| Repeats (every day of the week) |  |  |  |
| Repeats for (2 weeks or + 1 week) |  |  |  |
| Time (12.30pm) |  |  |  |
| Second Time (6pm)  (additional reminder should be set in Google Calendar) |  |  |  |

Sub total = /6

| **Assignment 2** | Correct? | Any notes about mistake, if made | Help needed? |
| --- | --- | --- | --- |
| Name (Jack’s birthday or similar) |  |  |  |
| Location (Café) |  |  |  |
| Date/when (as asked) |  |  |  |
| Time (12.30pm) |  |  |  |
| Repeats (none) |  |  |  |
| Notification (30 mins before) |  |  |  |

Sub total = /6

| **Assignment 3** | Correct? | Any notes about mistake, if made | Help needed? |
| --- | --- | --- | --- |
| Name (GP appointment) |  |  |  |
| Date (as asked) |  |  |  |
| Time (2pm) |  |  |  |
| Duration (30 mins) |  |  |  |
| Repetition (none) |  |  |  |
| Notification (at time of event) |  |  |  |
| Notes/Add description (repeat prescription) |  |  |  |

Sub total = /7

**Total =** /19 Pass mark = 13

Assignment 1

Starting on **this coming Monday** you are going to attend an Open University class.

These classes are every weekday at 12.30pm – 2.30pm.

They repeat for the next four weeks.

You will need to be reminded 1 hour before the event.

Set the following Appointment event:

Title: Open University Class

Date/when: **This coming Monday**

Time: 12.30pm

Duration: 2 hours

Repeats?: Mon, Tue, Wed, Thur, Fri. This week + 3 more weeks

Notification: 1 hour before

Assignment 2

You have made an appointment with your physio on **This coming Friday** between 2-2.30pm.

You will need to be reminded 1 hour before.

Make a note to mention your back pain during this appointment.

Set the following Appointment event:

Title: Physio

Date/when: **This coming Friday**

Time: 2pm

Duration: 30 mins

Repeats?: does not repeat

Notification: 1 hour before

Notes: Mention back pain

Assignment 3

Due to your Physio appointment you won’t be able to attend your Open University course **this coming Friday.**

Remind yourself of this in the morning.

Set the following Other reminder:

Title: No class today

Date/when: **This coming Friday**

Time: 10am

Repeats?: does not repeat

Notification: At time of event

| **Assignment 1** | Correct? | Any notes about mistake, if made | Help needed? |
| --- | --- | --- | --- |
| Name (Open university class or similar) |  |  |  |
| Date/when: (as asked) |  |  |  |
| Time (12:30) |  |  |  |
| Duration (2 hours) |  |  |  |
| Repetition (Mon-Fri - every weekday) |  |  |  |
| Repetition (for 4 weeks) |  |  |  |
| Notification (1 hour before) |  |  |  |

Sub total = /7

| **Assignment 2** | Correct? | Any notes about mistake, if made | Help needed? |
| --- | --- | --- | --- |
| Name (Physio or similar) |  |  |  |
| Date/when (as asked) |  |  |  |
| Time (2pm) |  |  |  |
| Duration (30 mins) |  |  |  |
| Repetition (none) |  |  |  |
| Notification (1 hour before) |  |  |  |
| Note (back pain or similar) |  |  |  |

Sub total = /7

| **Assignment 3** | Correct? | Any notes about mistake, if made | Help needed? |
| --- | --- | --- | --- |
| Name (No class today or similar) |  |  |  |
| Date (as asked) |  |  |  |
| Time (10am) |  |  |  |
| Repetition (none) |  |  |  |
| Notification (at time of event) |  |  |  |

Sub total = /5

**Total =** /19 Pass mark = 13
