# Supplementary material for: AppReminders – a pilot feasibility randomized controlled trial of a memory aid app for people with acquired brain injury
Source: Neuropsychol Rehabil. 2023 Jun 13;34(4):535–71. doi: 10.1080/09602011.2023.2220969 (PMC11166046; doi:10.1080/09602011.2023.2220969)
Supplement: supplimentary_materials_doc_1.docx [file PNRH_A_2220969_SM7177.docx]

# Supplementary files document 1

## ApplTree

Using co-design methods with prospective users, we developed ApplTree - a smartphone reminding application with design features that can be personalised to individuals. ApplTree prompts reminder setting, supports reminder entry to improve accuracy, and delivers users with prompts at the appropriate times. We designed ApplTree (Figures 1 and 2) with features that our research suggests can help support independent use and increase the efficacy of prompting technology interventions, and which can be personalised according to need and preference. One feature is unsolicited prompting (figure 1, top right). This prompts the user to consider if they need to set any reminders. This has been found to increase the number of reminders set when used by people with ABI (Jamieson et al., 2017; Jamieson et al., 2022(a)). Two other user interface design features aim to improve the usability of the app. Narrow-deep user interface design (figure 2 – right) is an input method that has many screens with a small amount of information on each. This contrasts with broad-shallow user interface design (figure 2 – left) that has a lot of information on fewer screens. There is evidence that narrow-deep design is easier to use for people with cognitive impairments (Hu et al., 2015). People with ABI miss significantly less information when setting reminders using a narrow deep interface when compared to broad-shallow design (Jamieson et al., 2022(b)). Finally, a decision tree user interface design is incorporated into the app. Figure 3 shows the opening screen and medication branch of this decision tree. The opening screen has six different ‘branches’ that can be selected – appointment, medication, social event, shopping, birthday and other). These diverse types of reminders require different pieces of information to be entered and each reminder has different branches of information. To develop the information needed for these six types of reminders we completed a co-design session with ten clinicians working with people with acquired brain injury and 3 service users at an inpatient rehabilitation unit in 2018.


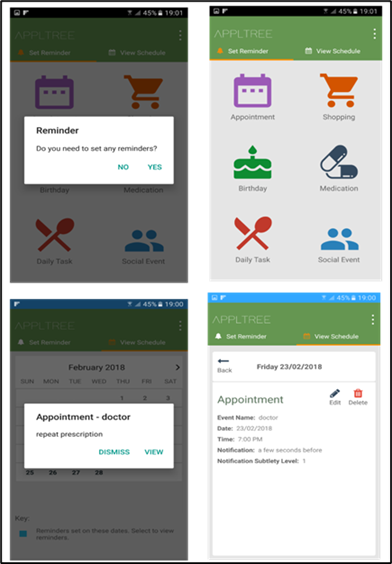


Figure 1. ApplTree unsolicited prompt (top left), opening screen (top right), an example of a reminder notification (bottom left), and example of an event summary (bottom right). The opening screen shows the six reminder types that each have different ‘branches’ of information when the decision tree feature is switched on.


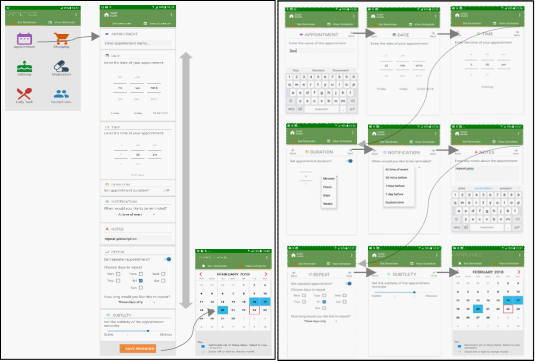


Figure 2. ApplTree Broad/Shallow UI (on the left) and ApplTree narrow/Deep user interface (on the right).


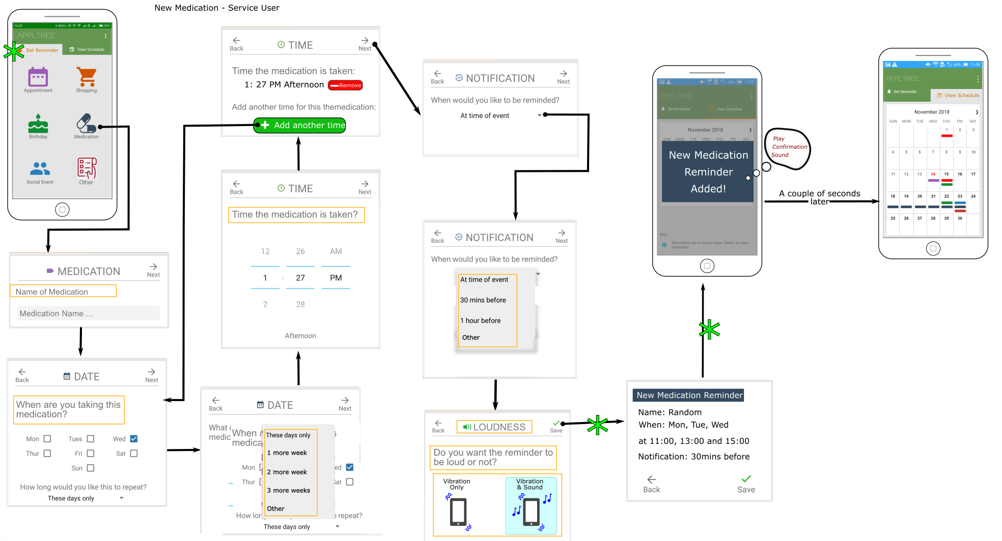


Figure 3. Decision tree user interface design – the ‘branch’ for a medication reminder in ApplTree.

## Google Calendar

Google Calendar is a reminding app freely available to download from app stores on both iOS and Android devices. It allows users to set events that have detailed information (e.g. time, date, repeat, location, notifications ahead of the event) and reminders or tasks with only title, date, time and repeat options. The opening screen shows the upcoming events the person has coming up and allows scrolling navigation through past and future events, tasks and reminders (figure 4 - left). It uses a broad-shallow user interface for setting the reminders. All information to be entered is presented on one screen and scrolling is necessary for navigation of information hidden in the initial screen (figure 4 - right). Google Calendar is in widespread use with over 1 billion downloads from the Google Play store).


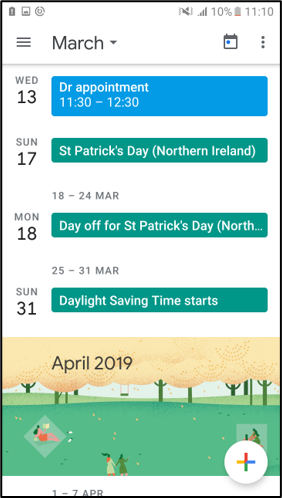

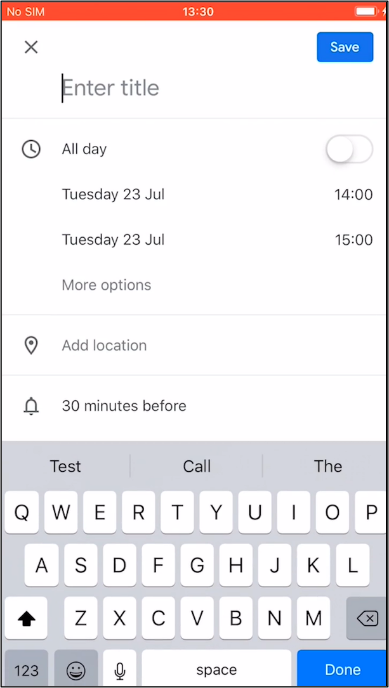


Figure 4. Opening screen (right) and Scrolling navigation for event entry (left) in Google Calendar (version for download in 2019)

# Memory logs and Text times

Memory log ring-binders were either given in-person or posted to participants’ home address prior to both the baseline and follow-up sessions. These folders consisted of 3 weeks’ worth of memory logs (1 sheet for each day, see appendix for example memory log sheet). Participants filled out memory logs with intended tasks for the week ahead during in-person or phone call sessions with researchers. An activities of everyday living checklist (see appendix) was used as a prompt if participants found it difficult to think of tasks they needed to remember. Eight memory task spaces were available for each day in the memory log (though more memory tasks could be added). For each memory task participants were asked to say when the task was for, and then each evening check off: 1) if the task had been completed, 2) if it was early, on time or late, 3) note if there was any reason (apart from them forgetting) that meant the task did not happen or was late (e.g. an appointment that got cancelled). Participants were asked to return the memory logs in person or by post using freepost envelopes provided. After the study began an extra item was introduced in the memory log – whether or not the participant needed reminded by someone about the task. This was then added in as an additional question although the primary outcome variable was still calculated using only the information about whether the task was completed and if it was on time.

Researchers reviewed the previous week’s memory log with participants during weekly calls in both baseline and follow-up phases. Two researchers completed all recruitment, baseline sessions and follow-up sessions in the study. One researcher conducted all participant randomisations and so was not blinded to condition. The other researcher was blinded to condition and completed all the follow-up memory log calls with participants. This blinded approach was intended to remove any potential bias involved in scoring the memory performance from the memory logs. A statement was read out by the blinded researcher before each follow-up call to remind participants not to discuss the app.

Memory logs were scored using both the completed memory logs returned to the research team and the researchers memory log noted from the call with the participant. The participant’s memory log was prioritised so it would be scored first, and any missing data was filled in using the researcher log. Where there was disparity between the researcher and participant logs, the participant log information was used

Text timesheets were included in the memory log ring-binders. Participants were asked to text a study mobile phone as close as possible to the four times provided for each day of the baseline and follow-up phase. The text time dates corresponded to the dates when participants completed the memory logs. Three sets of times were offered to participants depending on what suited them – early (times between 8am and 3pm), late (times between 12pm until 8pm) and in-between (times between 9am and 5pm). Participants were told to complete as many of the texts as possible, as close to the time as they could. They were told that it was not expected for them to send all the texts on time and that if they missed any texts they should not worry and move on to the next one. They were not given any instructions on strategies and could use any strategy they wanted to help remember the times. Texts were received by a study phone which automatically logged the time the text was sent. Participants were given a point for each text send between 30 minutes before and 30 minutes after the time for that day. For each day they could receive a maximum of 4 and a minimum of 0 points.

#### Memory log and text time scoring

1. Before summarising data, individual task records were excluded for one or more of the following reasons:
   1. if there was an external reason for the task not being performed
   2. if there was no data recorded on whether the task was performed or
   3. if there was no data recorded on whether the task was performed early, on time or late when data indicated the task was performed
2. Memory performance score was calculated for each task using both a3-point scale, and a 4-point scale where possible. The 3-point scale was used where participants began study prior to introduction of question on whether someone needed to prompt them and the 4-point scale was used where participants were asked whether someone needed to prompt them.
3. *3-point scale:*

| Score | Rule |
| --- | --- |
| 0 | Participant did not do the task |
| 1 | Participant did the task late |
| 2 | Participant did the task early or on time |

1. *4-point scale:*

| Score | Rule |
| --- | --- |
| 0 | Participant did not do the task |
| 1 | Participant did the task late and someone gave them a reminder |
| 2 | Participant did the task early or on time and someone gave a reminder  OR  Participant did the task late and nobody gave a reminder |
| 3 | Participant did the task early or on time and nobody gave a reminder |

1. Daily memory performance score for a participant = $(sum of all task memory performance scores in specific day$) $\div(total possible points to gain for all memory performance tasks in specific day)$

The higher the score, the better the participant’s memory performs. The participant’s average baseline and intervention phase scores were calculated by taking the mean of the daily scores within the appropriate study phase.

#### Comparing Memory Performance for the ApplTree and Google Calendar groups

Our pre-determined measure of memory ability performance score (on 3-pt scale) was calculated as follows:

1. Daily memory log score was calculated as the mean of each day’s recorded tasks, scored between 0 and 2.
2. Daily text time scores were calculated as the proportion of the 4 texts sent within 30 mins of allocated time.
3. A daily overall memory performance score was calculated by taking the mean of the respective day’s daily memory log score and text times score.
4. The average overall memory performance score (primary outcome) is calculated as the mean of the daily overall memory performance scores, separately for the days occurring in the baseline phase and intervention phase.

Descriptive were used to summarise differences in memory performance brought about by the interventions.  A two-sample t-test was used for the between-group comparison of the change in memory score.

**UTAUT (Unified Theory of Acceptance and Use of Technology) Questions/statements:**

Each statement is rated on a likert scale from 1-6; totally disagree = 1 -> totally agree = 6

Performance expectancy:

I find the app useful _____

Using the app enables me to accomplish more tasks_____

Using the app increases my productivity_____

Effort expectancy

My Interaction with the app is clear and understandable_____

It was easy for me to become skilful at using the app_____

I found the app easy to use_____

Learning to operate the app was easy for me______

Attitude toward using technology

Using the system is a good idea_____

The app makes setting a schedule more interesting_____

Using with the app is fun_____

I like using the app_____

Social influence

People who influence my behaviour think that I should use the app_____

People who are important to me think that I should use the app_____

Family and/or carers have been helpful in the use of the app_____

In general, the those around me have supported the use of the app_____

Facilitating conditions

I have the resources necessary to use the app_____

I have the knowledge necessary to use the app_____

The app is compatible with other systems I use_____

A specific person (or group) is available for assistance with app difficulties_____

Self-efficacy

I could set a reminder using the app if...

If there was no one around to tell me what to do as I go_____

If I could call someone for help if I got stuck_____

If I had a lot of time to complete the job for which the software was provided____

If I had just the built-in help facility for assistance_____

Anxiety [inverted scores]:

I feel apprehensive about using the app_____

Its cares me to think that I could lose a lot of information using the app by hitting the wrong key_____

I hesitate to use the app for fear of making mistakes I cannot correct_____

The app is somewhat intimidating to me_____

Behavioural intention to use the system

I intend to use the app in the next 6 months_____

I predict I will use the app in the next 6 months_____

I plan to use the app in the next 6 months_____
